# Multi-omic insights into the formation and evolution of a novel shell microstructure in oysters

**DOI:** 10.1186/s12915-023-01706-y

**Published:** 2023-09-29

**Authors:** Yitian Bai, Shikai Liu, Yiming Hu, Hong Yu, Lingfeng Kong, Chengxun Xu, Qi Li

**Affiliations:** 1 Key Laboratory of Mariculture, Ministry of Education, Ocean University of China, Qingdao, 266003 China; 2 Laboratory for Marine Fisheries Science and Food Production Processes, Qingdao National Laboratory for Marine Science and Technology, Qingdao, 266237 China

**Keywords:** Biomineralization, Oyster, Shell, Chalky layer, Genome, Pif evolution, Neo-functionalization

## Abstract

**Background:**

Molluscan shell, composed of a diverse range of architectures and microstructures, is a classic model system to study the relationships between molecular evolution and biomineralized structure formation. The shells of oysters differ from those of other molluscs by possessing a novel microstructure, chalky calcite, which facilitates adaptation to the sessile lifestyle. However, the genetic basis and evolutionary origin of this adaptive innovation remain largely unexplored.

**Results:**

We report the first whole-genome assembly and shell proteomes of the Iwagaki oyster *Crassostrea nippona*. Multi-omic integrative analyses revealed that independently expanded and co-opted tyrosinase, peroxidase, TIMP genes may contribute to the chalky layer formation in oysters. Comparisons with other molluscan shell proteomes imply that von Willebrand factor type A and chitin-binding domains are basic members of molluscan biomineralization toolkit. Genome-wide identification and analyses of these two domains in 19 metazoans enabled us to propose that the well-known Pif may share a common origin in the last common ancestor of Bilateria. Furthermore, Pif and LamG3 genes acquire new genetic function for shell mineralization in bivalves and the chalky calcite formation in oysters likely through a combination of gene duplication and domain reorganization.

**Conclusions:**

The spatial expression of SMP genes in the mantle and molecular evolution of Pif are potentially involved in regulation of the chalky calcite deposition, thereby shaping the high plasticity of the oyster shell to adapt to a sessile lifestyle. This study further highlights neo-functionalization as a crucial mechanism for the diversification of shell mineralization and microstructures in molluscs, which may be applied more widely for studies on the evolution of metazoan biomineralization.

**Supplementary Information:**

The online version contains supplementary material available at 10.1186/s12915-023-01706-y.

## Background

Biomineralized exoskeleton represents a key evolutionary innovation that contributes to the rapid diversification of living organisms dating back to the early Cambrian [1]. Among mineralizing metazoans, Mollusca particularly benefits from the various functions of mineralized shell [2], resulting in the evolutionary and ecological success of this extremely diverse phylum [3]. Molluscan shells are composed of calcium carbonate crystals and multiple organic matrix components comprising proteins, polysaccharides, and lipids [4]. In spite of a minor organic part in shell by mass, shell matrix proteins (SMPs) play critical roles in shell construction [5]. Over the past decades, a wide array of SMPs have been identified from molluscan shells [6]. These proteins are typically involved in the biomineral deposition, contributing not only to the formation of organic framework but also to the nucleation and growth of calcium carbonate polymorphs (calcite and aragonite) [7–9]. In addition, recent studies suggested that many SMPs are multifunctional, also participating in immunity and signaling processes [6, 10]. The multifunctionality of SMPs highlights their complex roles and evolutionary origins in molluscan biology.

Generally, molluscan shells contain multiple layers, and each layer is characterized by a specific shell microstructure (e.g., prismatic, crossed-lamellar, nacreous, and homogeneous) [11, 12]. Despite the conservation of shell morphology within many taxa, ultrastructural and molecular analyses have revealed that shell microstructures have evolved independently multiple times across various molluscan lineages [2, 13]. At the proteomic level, a distinct partitioning of SMPs has been observed among different shell microstructures [6, 14, 15]. Interestingly, similar shell microstructures in different molluscs are comprised of markedly distinct proteins [16, 17]. This suggests that SMPs have rapidly evolved in several molluscan lineages, leading to the diversification of shell microstructures. The molecular evolution of SMPs, driven by powerful mechanisms such as gene duplication, domain recruitment, and exon-shuffling, are often accompanied by the emergence of novel phenotypes of shell mineralization [18, 19]. Gene duplication for functional diversification (neo-functionalization or sub-functionalization) has been considered as a fundamental process for the acquisition of a novel gene encoding SMP [20]. However, the evolutionary relationships between SMPs and diverse shell microstructures remain largely unclear.

The bivalve family Ostreidae, commonly known as oyster, is the only one molluscan group employing chalky calcite as a shell layer [21–23]. The chalky layer, an autapomorphy of oysters, is distinct from the vesicular microstructure in Gryphaeidae, the sister family of Ostreidae [24]. The unique chalky microstructure plays a pivotal role in the mechanical properties and rapid growth of oyster shell [25–27]. Intriguingly, recent observations have indicated that chalky calcite deposits at the growth break of the oyster shell, suggesting that chalky microstructure maintains an advantageous internal space to allow the oyster to cement to an uneven substrate [27]. Such a novel shell microstructure proves particularly beneficial for the sessile lifestyle of oysters, providing them with a robust and flexible attachment mechanism. Previously, various SMPs have been identified and characterized in the whole shell of Pacific oyster *Crassostrea gigas*, using proteomic approaches [28–31]. These studies have significantly expanded our understanding of the complex protein composition of the oyster shell. However, only few studies have been performed on the SMPs of chalky layer in oysters [31]. Moreover, the studies on the SMPs of oyster have been mostly performed in the species *C. gigas.* The SMPs of other oyster species and their roles in the formation of chalky microstructure are not fully understood. Consequently, the molecular details of chalky layer formation remain enigmatic. Even more elusive is the evolutionary origin of the novel shell microstructure.

In this study, we focus on a member of Ostreidae family, the Iwagaki oyster *C. nippona*. Given the considerable deposition of chalky calcite in its left valve, *C. nippona* serves as an ideal model for studying shell mineralization. To better understand the genetic basis of chalky layer formation, we assembled a chromosome-level genome of *C. nippona* and generated comprehensive transcriptomic resources. In addition, proteomic analyses of three types of *C. nippona* shell layers (prismatic, foliated, and chalky layers) were constructed using liquid chromatography tandem mass spectrometry (LC–MS/MS). Comparative genomic and shell proteomic analyses allow us to explore molecular features of SMPs in oyster chalky layer, providing insights into the evolution of chalky microstructure and evolutionary conservation of shell microstructures. Our study also broadens the understanding of the origin and functional evolution of key genes underlying the diversity of molluscan biomineralization.

## Results and discussion

### Genome assembly and annotation

**Fig. 1:**
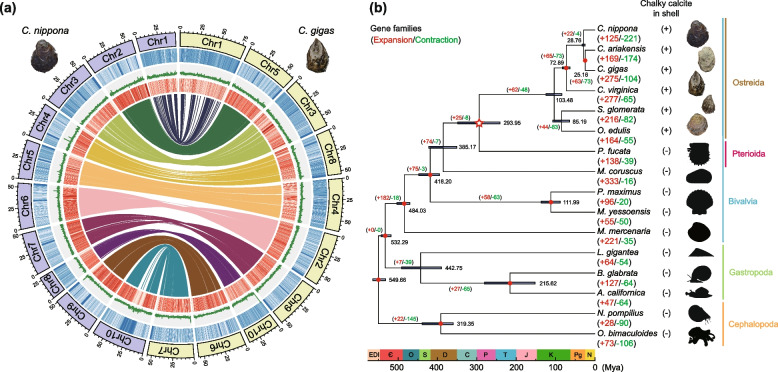
Genomic landscapes of two oysters and the distribution of chalky layer in molluscs. **a** Circos plots showing conserved synteny between *Crassostrea nippona* (left) and *C. gigas* (GenBank accession number: GCA_011032805.1) (right). From outer to inner circle: repeat coverage, GC content, gene distribution, genomic synteny. The sliding window size is 100 kb. **b** Phylogenetic tree of 16 representative molluscs. Nine nodes with red dots represent reference-calibrated time points (Additional file 2: Table S7). The node with a red hollow star shows the divergence time between Ostreida and Pterioida. The plus signs after species names indicate chalky calcite in the shell; minus signs mean no chalky calcite in the shell. The purple horizontal lines indicate the 95% confidence intervals of divergence times. Numbers of gene families undergoing expansion and contraction for each lineage are shown in red and green, respectively. The color labelling scheme of taxa: Bivalvia (blue), Gastropoda (green), Cephalopoda (orange), Ostreida (brown), Pterioida (purplish red). Periods: Cambrian (Є), Carboniferous (C), Devonian (D), Ediacaran (EDI), Jurassic (J), Cretaceous (K), Neogene (N), Ordovician (O), Permian (P), Paleogene (Pg), Silurian (S), Triassic (T)

A chromosome-scale genome assembly of *C. nippona* was constructed from 27.5 Gb (~ 67-fold coverage) of PacBio high-fidelity circular consensus sequencing (HiFi-CCS) reads and 60.8 Gb of Hi-C (high-throughput chromosome conformation capture) sequence data (Fig. 1a; Additional file 1: Fig. S1a). The assembly has a total length of 530.1 Mb (Scaffold N50 = 50.9 Mb) and a GC content of 33.8% (Additional file 2: Table S1). The assembled genome size was consistent with the estimation based on k-mer analysis (*k* = 17) (Additional file 1: Fig. S1b), and the result from flow cytometry analysis in a previous study [32]. The high quality of our genome assembly was supported by over 99% mapping rate of sequencing reads (Additional file 2: Table S1) and 97.3% of Benchmarking Universal Single-Copy Orthologs (BUSCO) completeness against the metazoan core gene set (Additional file 2: Table S2).

A large portion (44.69%) of the *C. nippona* genome was annotated as repetitive elements, which were dominated by DNA transposons (17.90%) and Helitrons (13.35%) (Additional file 1: Fig. S2, Additional file 2: Table S3). In total, 28,871 protein-coding genes were predicted in the *C. nippona* genome, with an average gene length of 9005 bp (Additional file 2: Table S4). Approximately 96% of predicted genes were functionally annotated using various public databases (Additional file 2: Table S5). Assessment of the predicted genes using BUSCO analysis revealed that 94.8% of the predicted genes were complete ortholog genes (Additional file 2: Table S2). In addition, a total of 5401 non-coding RNA (ncRNA) genes were identified in the *C. nippona* genome, including 2474 tRNAs, 1847 rRNAs, 1036 snRNAs, and 44 miRNAs (Additional file 2: Table S6).

### Oyster genome evolution and phylogenetic analysis

We found a high level of one-to-one syntenic relationship at the chromosome level between *C. nippona* and *C. gigas* (Fig. 1a). The high genomic collinearity was also observed among *Crassostrea* species (Additional file 1: Fig. S3a) and *Ostrea* oysters [33, 34]. Even though the lack of chromosome-scale genomes of many other oyster species, the high chromosome relatedness among *Crassostrea* and *Ostrea* genomes indicated the genomic conservation within oysters. Moreover, various inter-chromosomal rearrangements were observed between *C. nippona* and *Mizuhopecten yessoensis* (Additional file 1: Fig. S3b). The *M. yessoensis* was reported to possess a highly conserved 19-chromosome karyotype similar to that of the bilaterian ancestor [35]. The 10-chromosome karyotype of *C. nippona* was mainly derived from fusions of two or more ancestral chromosome segments of *M. yessoensis*, except for Chr1, which was only originated from an ancestral chromosome (Additional file 1: Fig. S3b)*.* Similar chromosome rearrangements were also observed between *C. nippona* and *Pinctada fucata* (Additional file 1: Fig. S3b)*.* Together, a combination of fission, fusion, and retention from the ancestral chromosomes resulted in the 10 chromosomes of oysters.

We selected 15 molluscan species with whole-genome assembly for comparative genomic analyses with *C. nippona* (Additional file 2: Table S2). A total of 1253 one-to-one single-copy orthologous genes were identified and used for the construction of phylogenetic tree (Fig. 1b, Additional file 2: Table S7). Molecular clock analysis based on the secondary calibrations suggested that the common ancestor of *C. nippona* diverged from *C. gigas* and *C. ariakensis* at 28.76 million years ago (Mya) (25.37–32.76 Mya) (Fig. 1b), in agreement with evidence from a previous study [36]. Within Bivalvia, the Ostreida and Pterioida diverged at 293.95 Mya (241.02–348.46 Mya) (Fig. 1b). The divergence time between Ostreida and Pterioida was inferred with the genomes of Pteriidae and Ostreidae species (Additional file 2: Table S2). Thus, the absence of genomic data of species in other order(s) of Ostreida (Grypheidae) or Pterioida (Malleidae, Isognomonidae, Pulvinitidae) may contribute to the high confidence interval of this node during divergence estimation. The fossil data have shown that Ostreida may originate from 279 to 265 Mya, which is closed to our estimation [36]. In addition, given the origin and the early divergence of Ostreida species [36, 37], our result supports the hypothesis that the Permian was a key period for the radiation of bivalves, and molluscan ecological dominance first occurred prior to the end-Permian mass extinction [38]. In a previous study, the Ostreidae was speculated to have originated ~ 255 Mya during the Permian–Triassic boundary [36]. The chalky calcite microstructure has been found in the shell layers of *Ostrea*, *Crassostrea*, and *Saccostrea* oysters [5, 21, 23, 39, 40]. Therefore, we speculated that this novel shell microstructure may originate around the end of the Permian period and result from the independent evolution in oysters. Global environmental change at the end of the Permian Period disturbed the diversity of biomineralization [41]. All biomineralizing cnidarians, along with the majority of brachiopod and stalked echinoderm species, disappeared at the end of the Permian Period, which might be caused by ocean acidification [41, 42]. The common ancestor of Ostreidae species may have to respond in distinct ways (e.g., chalky deposition in the shell) to selective pressure at the end-Permian mass extinction.

Among the selected genomes, we identified a set of 620 Ostreidae-specific gene families and 62 expanded gene families, respectively. Gene Ontology (GO) enrichment analyses of these gene families revealed the components involved in shell mineralization of oysters such as cell adhesion, extracellular matrix, fibronectin binding, and chitin binding (Additional file 2: Tables S8, S9). Moreover, gene families known to produce proteins involved in shell formation, such as tyrosinase [43], peroxidase [44], and tissue inhibitor of metalloproteinase (TIMP) [45], have undergone large independent expansions in Ostreidae (Additional file 1: Figs. S4a, S5a, S6a). The majority of these gene members were highly expressed in the mantle of *C. nippona* (Additional file 1: Figs. S4b, S5b, S6b, S7). Combined with shell proteomes of *C. nippona* (Additional file 2: Table S10), some of oyster-specific gene members were classified as SMPs in the chalky layer (Additional file 1: Figs. S4, S5, S6), suggesting their co-option into the evolution of the chalky calcite in the oyster shells.

### Evolution and formation of chalky calcite in oysters

**Fig. 2:**
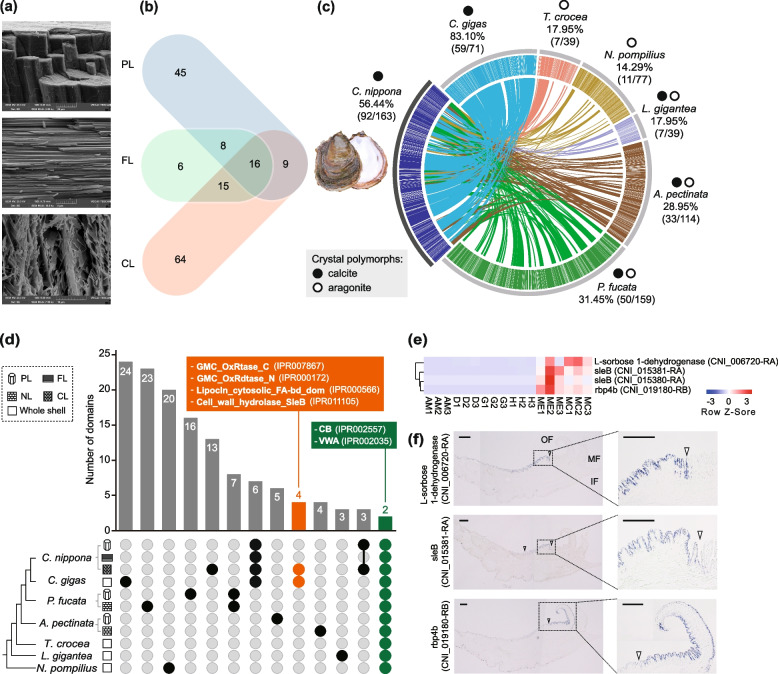
Microstructures and proteomes of the *Crassotrea nippona* shell. Shell layers: prismatic layer (PL), foliated layer (FL), chalky layer (CL), nacre layer (NL). **a** SEM micrographs of the microstructures of the prismatic, foliated, and chalky layers of the *C. nippona* shell. **b** Number of proteins identified from the prismatic, foliated, and chalky layers. **c** Circos diagram of seven representative molluscan shell proteomes (the *E*-value cut-off of BLASTP is 1e − 20); with the shell of farmed *C. nippona* on the left. Different color showed the sequences of SMPs in different molluscs and their proteins pairs with SMPs of *C. nippona.* Percentages and proportions in brackets indicate the number of SMPs having similarities between *C. nippona* and the other six molluscs. The solid circles above the species names represent that the shells contain calcite, while the hollow circles indicate the shells contain aragonite. **d** Upset plot comparing the protein domains identified from the shell proteomes of seven molluscs. The bar chart indicates the number of functional domains conserved among shell microstructure(s) or the whole shell of specie(s). The colored dots below histograms indicate the presence of the domains in shells of the molluscs. Domains only detected in the *C. gigas* shell and the chalky layer of *C. nippona* shell are indicated in orange. Domains shared by all shell layers across seven molluscs are colored in green. The complete results are shown in supplementary figure S11 (Additional file 1). **e** Expression of SMPs with domains specifically detected in *C. gigas* and the chalky layer of *C. nippona* in six tissues of *C. nippona*. Tissues: adductor muscle (AM), digestive gland (D), gill (G), hemolymph (H), mantle edge (ME), central mantle (MC). The number after the abbreviation of tissue represents biologically independent individuals (*N* = 3). **f** Spatial expression patterns of three SMPs with domains specific for chalky layers in the mantle tissue of *C. nippona*. The protein domain structures of genes are shown in the supplementary figure S12 (Additional file 1). Full view (left) and partial enlargement (right) show positive cells stained in blue by in situ hybridization of each gene, respectively. White arrows symbolize the end of the gene expression region. Mantle folds: outer fold (OF), middle fold (MF), inner fold (IF) (scale bars: 200 µm)

Like other oyster species [5, 21, 23, 39, 40, 46], *C. nippona* has a pure calcite shell, with an outer layer of calcite prisms and inner multi-layered structures consisting of repeated foliated and chalky layers (Fig. 2a, Additional file 1: Fig. S8). In the multi-layer, the foliated layer is stacked by dense sheets of folia, while chalky structures are constituted of loose calcite blades with ample interconnected porosity (Fig. 2a). Foliated calcite is a common microstructure in many pteriomorphian bivalves [47], whereas chalk is found exclusively in oysters [21–24]. The diversity of shell microstructures is usually associated with life habits [47]. Oyster has a sessile lifestyle supported by cemented attachment. The shapes of oyster shells are often influenced by the attached surfaces [27]. The chalky calcite deposition enables oyster shells, particularly the left valves, with a high degree of morphological plasticity in shape, allowing oysters to conform to irregular substrates in estuarine or intertidal zones while maintaining a favorable internal space [27]. The chalky layer represents a unique evolutionary innovation in oysters, facilitating their adaptation to a sessile life. In addition, chalky microstructure prevents the crack propagation of the oyster shell, which may serve a similar function to the holes in bones [48], implying the potential convergent evolution of biomineralized skeletons in oysters and vertebrates.

A specific mutation in an ELC protein has been identified as a key factor influencing the formation of chalky microstructure in *C. gigas* [20]. However, SMPs are elements of a comprehensive regulatory network and work cooperatively to form a given microstructure [6]. The production of shell microstructure cannot be solely attributed to certain separated constituents. To comprehensively investigate the molecular basis of chalky layer formation, we performed proteome sequencing of the prismatic, foliated, and chalky layers from the *C. nippona* shell and identified a total of 78, 45, and 104 SMPs, respectively (Fig. 2b, Additional file 1: Fig. S9, Additional file 2: Table S10). SMPs are secreted by the epithelial cells on the dorsal region of mantle. Expression patterns of genes encoding SMPs showed that most of these genes (82.2%) were highly expressed in the mantle (Additional file 1: Fig. S10). Comparative shell proteomic analysis indicated that 92 of *C. nippona* SMPs (56.4%) shared similarity with sequences in shell proteomes of the other six molluscs, including four bivalves, one gastropod, and one cephalopod (Fig. 2c). A high degree of unique matches was found between *C. nippona* and *C. gigas*, and the least number of matches was observed between *C. nippona* and *Nautilus pompilius* (Fig. 2c). The results are not only consistent with evolutionary divergence times, but also attributed to the crystal polymorphs of the shells of different species, as the shells of *C. nippona* and *C. gigas* are entirely calcite [21], whereas the *N. pompilius* shell is completely aragonite [49].

Further protein domain analysis was performed across SMPs from different shell microstructures of seven molluscs (Fig. 2d, Additional file 1: Fig. S11). Four functional domains were observed in both the *C. gigas* shell and the chalky layer of the *C. nippona* shell, including glucose-methanol-choline (GMC) oxidoreductase C-terminal, GMC oxidoreductase N-terminal, lipocalin/cytosolic fatty-acid binding, and cell wall hydrolase SleB (Fig. 2d). We speculated that these domains may represent a unique repertoire occurred exclusively in the oyster chalky layer. Four SMP genes containing the unique domains were highly expressed in the mantle of *C. nippona* (Fig. 2e, Additional file 1: Fig. S12). All these SMPs contained the signal peptide domain (Additional file 1: Fig. S12), suggesting that they were secreted by the outer mantle cells and potentially participated in the formation of chalky layer. These results implied that protein domain novelties may contribute to the formation and evolution of chalky microstructure. Notably, a previous study identified the GMC oxidoreductase domain unique to *C. gigas* by comparing shell proteomes of bivalves [10]. The GMC domain was discussed as the roles in the development, immunity, and chemical defense of insects [50–52]. However, the molecular function of GMC domain has remained largely unknown in molluscs. Similarly, the actual roles of lipocalin/cytosolic fatty-acid binding domain and cell wall hydrolase SleB domain still require further characterization in molluscan species.

### Mantle regions controlling the chalky layer formation

The mantle of molluscs is divided into different morphogenetic regions responsible for the secretion of SMPs that regulate the formation of specific shell layers [14, 18, 53]. For instance, the production of prismatic layer is controlled by SMP genes expressed in the outer zone of the mantle (outer pallium and mantle edge), while the nacreous layer-related SMP genes are expressed in the inner zone of the mantle (inner pallium) [14]. However, very little is known about the region of oyster mantle controlling the chalky layer formation. To identify the mantle regions associated with the production of chalky calcite, three highly expressed genes containing oyster-specific SMP domains, which were identified from seven molluscan shell proteomes (Fig. 2d), in the mantle of *C. nippona* (*L-sorbose 1-dehydrogenase*, *rbp4b*, and one of *sleB* genes) were selected to analyze their spatial expression in the mantle tissue (Fig. 2f). Interestingly, these genes showed different spatial expression patterns in the mantle. On the one hand, the signals of *L-sorbose 1-dehydrogenase* and *sleB* were observed in the dorsal regions of the inner pallium. On the other hand, *rbp4b* was expressed in two regions: the outer zone of the mantle as well as in the inner surface of the outer fold, which is involved in periostracum formation. The behavior of the mantle influences the formation of chalky layer during shell growth [27]. The presence of chalky deposition was observed outside of a *C. gigas* shell, between the periostracum and the substrate [54]. Therefore, the process of chalky layer formation may be flexible and co-regulated by multiple regions of the dorsal mantle epithelium, which may also be useful for the sessile life of oysters.

**Fig. 3:**
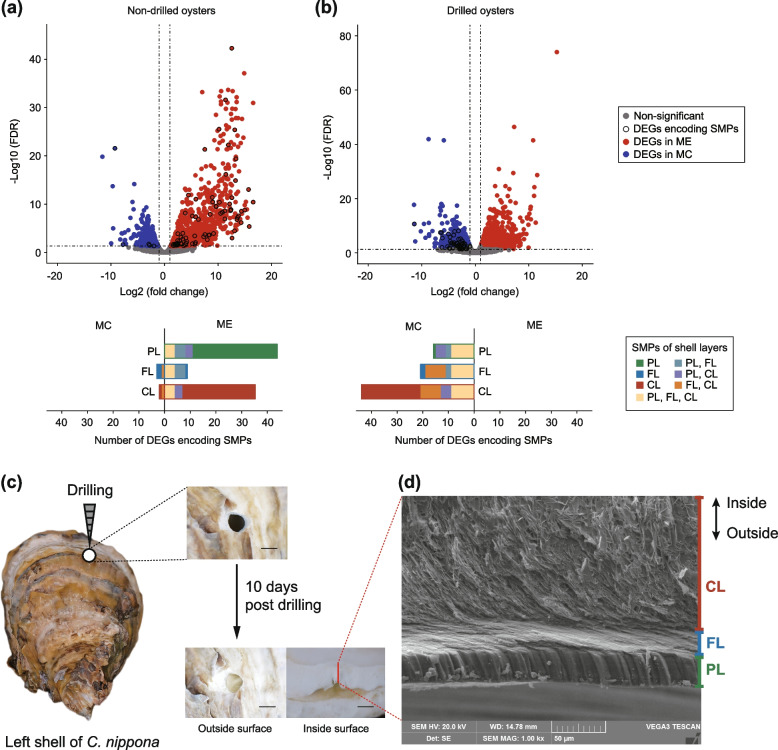
Shell reconstruction of *Crassostrea nippona*. **a** Differential gene expression and number of DEGs encoding SMPs in mantle tissues of non-drilled *C. nippona* and **b** drilled oysters. **c** Schematic illustration of shell-drilling experiment and observation of shell repair process of *C. nippona*. The red line on the bottom-right image indicates the location of the cross-sectional cut of repaired shell in **d** (scale bars: 5 mm). **d** Ultrastructure of cross section of the whole repaired shell. Shell layers: prismatic layer (PL), foliated layer (FL), chalky layer (CL)

Shell damage has been used to stimulate biomineralization in previous studies [55, 56]. To further explore the roles of distinct mantle regions in the chalky layer formation, we compared gene expression patterns in mantle edge (ME) and mantle center (MC) from the shell-drilled *C. nippona* and non-drilled individuals, respectively (Fig. 3, Additional file 1: Fig. S13, Additional file 2: Tables S11, S12). Notably, most of differentially expressed genes (DEGs) encoding SMPs of chalky layers were upregulated in the ME of the non-drilled oysters (Fig. 3a, Additional file 2: Table S11), whereas DEGs encoding SMPs of chalky layers were highly expressed in the MC during shell repair (Fig. 3b, Additional file 2: Table S12). An abundance of chalky calcite was deposited following the foliated layer in repaired shells (Fig. 3c, d, Additional file 1: Fig. S14), suggesting an active role of chalky deposition in shell repair. These results indicated that chalky layer formation was dominated by different mantle regions (ME or MC) under distinct physiological conditions of oyster (normal or shell repair condition), respectively. A recent study on ocean acidification proposed that chalky layer was produced by the mantle pallial of oysters [57]. This observation might only represent the regulation of chalky deposition by mantle pallial under low pH conditions. Our findings showed that chalky layer formation is flexibly controlled by oyster mantle regions (ME or MC), and the detailed regulatory mechanisms of SMP secretion and chalky calcite deposition still awaits further investigation. In addition, chalky microstructure was considered to be mediated by sulfate-reducing bacteria in some previous studies [58, 59]. However, the hypothesis was not supported by our molecular data and result that the chalky microstructure formation is controlled by the oyster mantles. The recent study by de Winter et al. (2021) also provided the isotopic and trace element evidence against formation of the chalky calcite by microorganisms [60]. Therefore, the chalky layer formation is biomineralization controlled by the oyster during shell growth, rather than microbially assisted mineralization.

### Evolution of Pif proteins involved in the diversification of shell microstructures

A biomineralization toolkit containing conserved domains within the Bivalvia was well-studied using shell proteomes or mantle transcriptomes [10, 56]. Although it was unclear about the detailed functions of these domains, the toolkit represented the core requirements for shell biomineralization. In our study, only two domains were completely conserved in the SMPs from different shell microstructures across molluscs (Fig. 2d). These domains were chitin-binding (CB) and von Willebrand factor type A (VWA), which were also classified as the members of molluscan biomineralization toolkit in previous studies [10, 15, 30, 49, 56, 61]. Based on these two domains, various SMPs were clustered into an orthogroup (OG0000000) which was shared by the shell proteomes of seven molluscs (Additional file 1: Fig. S15, Additional file 2: Table S13). These evidences point to the possibility that CB and VWA domains play crucial roles not only in the evolutionary conservation of shell microstructures, but also as the ancestral and basic components of molluscan biomineralization toolkit.

The VWA domain is often found in extracellular matrix proteins and has an adhesion function through protein–protein interaction [62]. The CB domain exhibits the high binding affinity to chitin and plays a critical role in the construction of various biomineralized exoskeletons [63–67]. Chitin is one of the major polysaccharides comprising the calcified shells of molluscs [7]. A chitinous scaffold provides the basic framework for interactions between extracellular matrix and calcium carbonates [68, 69]. CB and VWA domains have both been found to participate in chitin-scaffolding and arranging calcium carbonate crystals of molluscan shell [64, 68]. Thus, the combinations of VWA and CB domains may be associated with the diversity of shell microstructures.

**Fig. 4:**
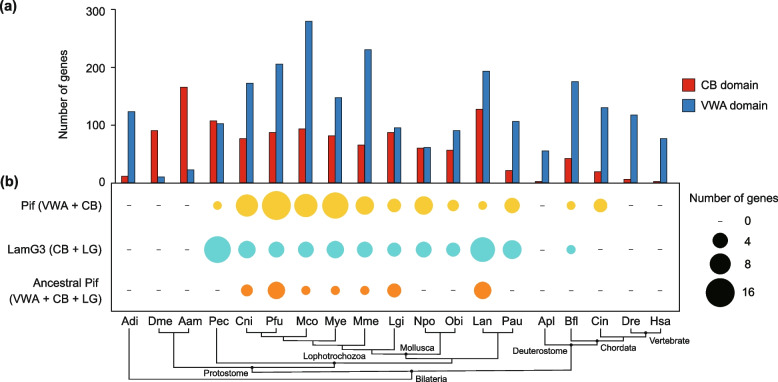
Distribution and combinations of chitin-binding (CB) and von Willebrand factor type A (VWA) domain-containing genes among 19 metazoans. Numbers of CB and VWA domain-containing genes (histogram graph in **a**) as well as Pif, ancestral Pif, and LamG3 genes (bubble chart in **b**) were determined in 19 metazoan genomes (Aam, *Amphibalanus amphitrite*; Adi, *Acropora digitifera*; Apl, *Acanthaster planci*; Bfl, *Branchiostoma floridae*; Cin, *Ciona intestinalis*; Cni, *Crassostrea nippona*; Dme, *Drosophila melanogaster*; Dre, *Danio rerio*; Hsa, *Homo sapiens*; Lan, *Lingula anatina*; Lgi, *Lottia gigantea*; Mco, *Mytilus coruscus*; Mye, *Mizuhopecten yessoensis*; Mme, *Mercenaria mercenaria*; Npo, *Nautilus pompilius*; Obi, *Octopus bimaculoides*; Pau; *Phoronis australis*; Pec, *Paraescarpia echinospica*; Pfu, *Pinctada fucata*). The schematized phylogenetic tree below the bubble chart indicates the evolutionary relationships among species

To further investigate the potential contribution of conserved CB and VWA domains to the evolution of chalky microstructures and molluscan shell biomineralization, we identified the distribution and combination of these domains in the genome databases of 19 metazoans (Fig. 4). The results demonstrated that CB and VWA domains are widely distributed in diverse metazoan lineages (Fig. 4a). Notably, the well-known combination of VWA and CB domains, Pif protein, was found commonly in many metazoan genomes (Fig. 4b). This acidic matrix protein and its homologs were not only identified in molluscan shells [14, 30, 70, 71], but also involved in the construction of other mineralized structures of molluscs, such as sclerites and shell-like eggcase [72, 73]. Despite VWA and CB domains are common across metazoans, we found that Pif proteins were exclusively present in lophotrochozoans and two chordates (*Branchiostoma floridae* and *Ciona intestinalis*) (Fig. 4b). The conserved domain architecture with VWA, CB, and concanavalin A-like lectin/glucanase (LG) domains was previously considered as an ancestral Pif, which occurred in the last common ancestor (LCA) of Mollusca and Brachiopoda [30, 71]. While in our study, the Pif proteins were not only observed in the genomes of bivalves, gastropods, and the brachiopod *Lingula anatina*, but also in other lophotrochozoans and chordates (Fig. 4b). Interestingly, Pif and its homologs, LamG3 proteins, were uncovered in the SMPs of *C. nippona* (Additional file 1: Fig. S16). The LamG3 proteins, composed of both CB and LG domains yet no VWA domain, were also present in lophotrochozoans and *B. floridae* (Fig. 4b). Taken together, LamG3, Pif, and ancestral Pif shared similar structures and distribution in metazoans.

**Fig. 5:**
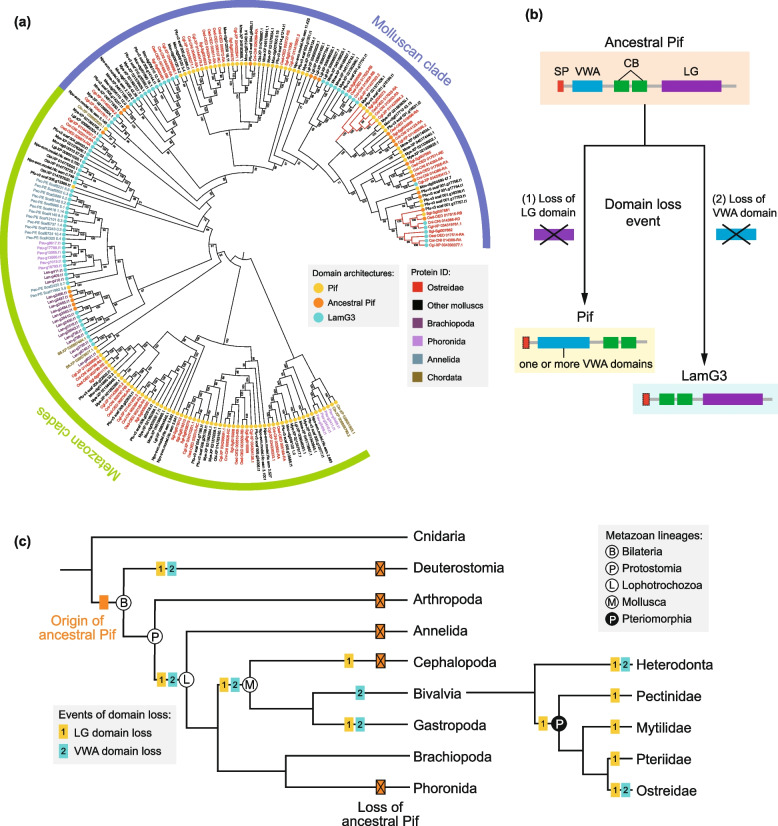
Evolution of Pif and LamG3 in metazoans. **a** Maximum likelihood (ML) tree of Pif and LamG3 in 16 selected metazoans. The different colors of protein IDs represent distinct lineages. Yellow, orange, and blue solid circles before protein IDs represent Pif, ancestral Pif, and LamG3 genes, respectively. Red branches on the clades represent three groups of genes which are all highly expressed in the mantle tissue (Additional file 1: S17). Numbers on the nodes are bootstrap values (> 50%). **b** The evolutionary model of LamG3, Pif, and ancestral Pif. Red box represents the signal peptide (SP) domain. Blue, green, and purple boxes represent chitin binding (CB), von Willebrand factor type A (VWA), and concanavalin A-like lectin/glucanase (LG) domains, respectively. Dash box indicate potential presence of SP domain. **c** Reconstructions of evolution of Pif and LamG3 in metazoans. Orange boxes on the branches indicate the origin or loss of ancestral Pif genes. Yellow and blue boxes represent the events of VWA and LG domain loss, respectively

To understand the evolutionary relationships of Pif and LamG3, a molecular phylogenetic tree was constructed using Pif, ancestral Pif, and LamG3 proteins from 16 metazoans (Fig. 5a, Additional file 2: Table S14). Notably, the lophotrochozoan Pif and LamG3 genes formed the metazoan clades with those of chordates (Fig. 5a), suggesting that the evolutionary origin of Pif genes is prior to the split of deuterostomes and protostomes. Interestingly, a large amount of Pif and LamG3 genes of molluscs formed the separate molluscan clade from other metazoans (Fig. 5a). In addition, most of these genes were highly expressed in the mantle of molluscs (Additional file 2: Fig. S17). Particularly, three groups of genes in molluscan clade were all highly expressed in the mantle tissues of bivalves (Fig. 5a). These results implied that the three groups of Pif and LamG3 genes have potential functions in shell biomineralization. However, many Pif and LamG3 genes did not exhibit high expression level in shell-forming mantle tissues of molluscs (Additional file 2: Fig. S17). Previous studies have indicated that no Pif proteins existed in the shell proteomes of brachiopods [63, 74, 75] and tube proteome of *Paraescarpia echinospica* [67]. Our findings are consistent with the hypothesis that the ancestral function of Pif may be not related to biomineralization [30]. Thus, Pif and LamG3 were independently co-opted for shell biomineralization in the molluscan lineages.

We further proposed the evolutionary model and scenario of Pif, ancestral Pif, and LamG3 (Fig. 5b, c). Briefly, the ancestral Pif underwent the domain loss of VWA and LG, resulting in generation of LamG3 and Pif, respectively (Fig. 5b). The molecular phylogenetic analysis indicated that Pif and LamG3 had a common origin in Bilateria (Fig. 5a, c). However, different major clades may diverge from multiple copies of the ancestral Pif in the LCA of Bilateria (Fig. 5a). Although our analysis was based on the genomes of a limited number of species, the results indicated that the Pif and LamG3 genes in Lophotrochozoa and Deuterostomia convergently evolved from the ancestral Pif (Fig. 5c). In addition, the ancestral Pif gene was preserved in Bivalvia, Gastropoda, and Brachiopoda, but lost in other lophotrochozoans as well as Arthropoda and Deuterostomia. Notably, VWA or LG domain loss events have occurred multiple times in the evolutionary process of molluscan lineages (Fig. 5c). The rapid evolution of the mantle secretome is essential in shell formation and diversity [16, 19]. The independent co-option of Pif and LamG3 genes for mantle-specific functions may be important driving forces acting on molluscan shell biomineralization and underlie the inter-specific differences observed in shell microstructures [18].

### Neo-functionalization of Pif and LamG3 genes facilitate the evolution of chalky microstructure

**Fig. 6:**
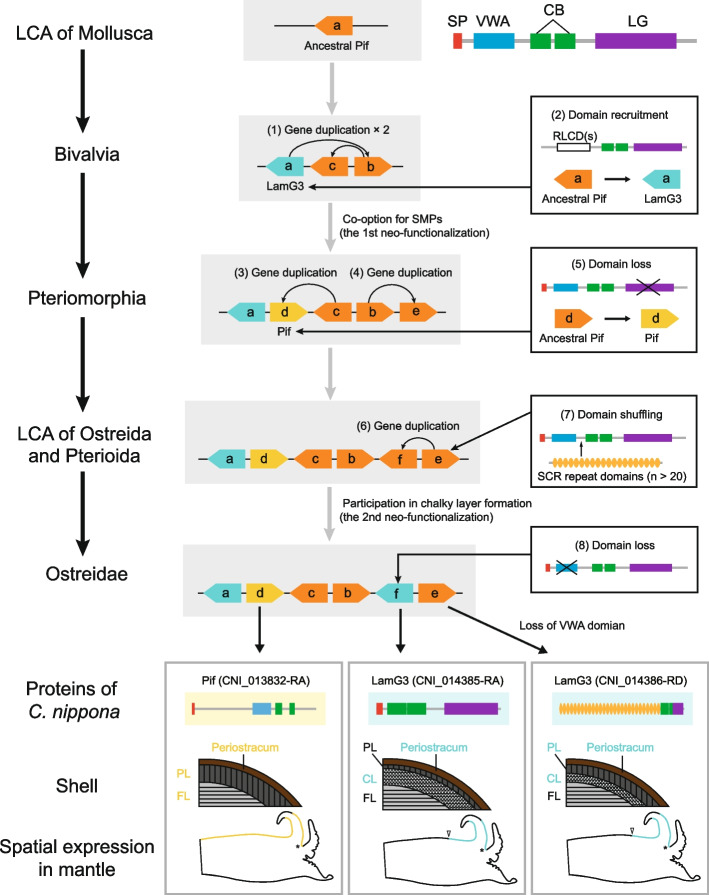
Evolution of the Pif_LamG3_cluster in molluscs. Gray background boxes show the gene cluster of Pif and LamG3. Right squares show the molecular evolution of gene structure. Numbers in brackets indicated the order of molecular evolution events. Asterisks indicate the periostracal groove. Yellow color in the representative model of mantle indicates the expression regions of Pif (CNI_013832-RA), while blue color indicates the expression regions of LamG3 (CNI_014385-RA and CNI_014386-RD). The detailed information of spatial expression patterns of the three genes in the mantle tissue of *C. nippona* have been shown in Figure S20 (Additional file 1). White arrows symbolize the end of the gene expression region. The names of shell layers which SMPs involved in are marked with colors in different genes (yellow for Pif, blue for LamG3). Shell layers: prismatic layer (PL), foliated layer (FL), chalky layer (CL). RLCD(s) means the repetitive low complexity domain(s)

Strikingly, an Ostreidae-specific LamG3 was identified as the SMP in the chalky layer of *C. nippona* (Fig. 5a, Additional file 1: Fig. S16). Deep mining of the oyster genomes revealed that the Ostreidae-specific LamG3, as well as a set of LamG3 and Pif genes, were clustered into a clade (Fig. 5a) and localized across the same chromosome (Additional file 1: Fig. S18). Therefore, we concluded with a hypothesized evolutionary process of origin and functionalization of the Pif_LamG3_cluster in bivalves (Fig. 6). This gene cluster was evolved from a single copy of ancestral Pif gene (Pif-a) in the LCA of Mollusca. Firstly, two successive reverse tandem duplication of the ancestral Pif gene occurred in Bivalvia, and the region encoding the C-terminal VWA domain in the Pif-a gene was deleted by domain recruitment. Then, two reverse tandem duplications of the ancestral Pif genes resulted in the generation of Pif-d and Pif-e in Pteriomorphia, respectively. In the LCA of Ostreida and Pterioida, Pif-f evolved from a reverse tandem duplication of Pif-e gene, but was lost in Pterioida. Afterwards, the Pif-e gene gained SCR repeat domain by domain shuffling. Finally, VWA domain loss of Pif-f gene resulted in the LamG3 in the LCA of Ostreidae, in which the chalky microstructure appeared in the shells.

The Pif-d and Pif-e emerged accompanying the functional evolution that manifested in their high expression level in bivalve mantles (Additional file 1: Fig. S19) and co-option as SMPs in the *C. nippona* shell (Additional file 1: Fig. S16). The spatial expression of these duplicated genes also indicated their novel function in shell biomineralization (Fig. 6, Additional file 1: Fig. S20). The Pif-d gene lost the sequence encoding the N-terminal LG domain and was expressed in the outer epithelial cells of the ME and MC (Fig. 6, Additional file 1: Fig. S20a, b). The Pif-d protein was identified in the prismatic and foliated layers of *C. nippona* (Fig. 6), thereby supporting that the Pif-d gene was involved in the formation of prismatic and foliated layers. In addition, the Pif-e gene in *C. nippona* underwent the domain loss of VWA domain and encoded an SMP involved in the prismatic and chalky layers (Fig. 6)*.* The Pif-e gene and its homolog, Ostreidae-specific LamG3 gene (Pif-f), were expressed in the dorsal surface of the mantle edge epithelium of *C. nippona* (Fig. 6, Additional file 1: Fig. S20c, d, e, f). The Ostreidae-specific LamG3 (Pif-f) lost the VWA domain and evolved as the SMP in the chalky layer (Fig. 6), suggesting that this gene acquired the novel function on the formation of chalky layer which may be secreted by the epithelium cells of ME. Interestingly, the three novel genes were all expressed in the inner surface of the outer fold (Fig. 6, Additional file 1: Fig. S20), which was responsible for periostracum synthesis [76]. Periostracum is a tanned organic membrane which is shared by Bivalvia and Gastropoda [77]. Hence, the conversed function of the ancestral Pif gene could be associated with the formation of periostracum in Bivalvia. In summary, Pif genes underwent two neo-functionalization events which contributed to shell formation and the chalky microstructure formation, respectively (Fig. 6).

Gene duplication from the parental copy usually results in functionally redundant genes that are not stably retained in the genome. In our study, although the duplicated genes were originated commonly from an ancestral Pif, distinct domain architectures enabled them to acquire novel functions involved in the formation of shell microstructures. Such functional evolution of Pif and LamG3 genes may be crucial for their simultaneous retention in the genome. In addition, the spatial expression patterns of these novel genes may be regulated by certain mutation(s) in the gene regulatory region [20]. Therefore, understanding evolutionary process of Pif_LamG3_cluster allows us to gain insights into the important role of gene duplication followed by functional diversification in acquiring evolutionary innovation for environmental adaptation.

## Conclusions

Oyster have evolved numerous innovations which poise them as remarkably successful reef builders and adaptors to sessile lifestyle. Emerging and diversifying over approximately 255 Mya, they are now distributed in marine ecosystems worldwide. Here, we present the first high-quality chromosome-level reference genome and shell proteomes for the Iwagaki oyster *C. nippona.* Our multi-omic analyses reveal that the independent expansion and co-option of genes are the key molecular innovations driving the formation and evolution of a novel shell microstructure, chalky calcite. Oysters have the ability to regulate the secretion of the chalky layer flexibly with distinct regions of the dorsal mantle epithelium under different conditions. This behavior may allow the oyster shell to exhibit a high degree of plasticity, facilitating the adaptation of oysters to a sessile life. Gene duplication and the dynamic combinations of conserved VWA and CB domains enable Pif genes to acquire novel functions for molluscan shell biomineralization and chalky microstructure formation in oysters. Our findings supported that key genetic components involved in biomineralization have evolved repeatedly from ancestral gene(s) without biomineral function. This study not only provides valuable insights to deeply understand the origin and evolutionary dynamics of molluscan shell biomineralization, but also paves the way for future research on biomimetic mineralization and novel material design.

## Methods

### Sample collection and genome sequencing

Multiple wild *C. nippona* individuals were collected from Zhoushan, Donghai Sea, China. The oysters were identified on the basis of both DNA fragments of cytochrome oxidase I (COI) and morphological observation. The samples were dissected, immediately frozen in liquid nitrogen, and stored at − 80 ℃ for further analysis. Genomic DNA was extracted from the adductor muscle of a male individual (674.3 g) by the standard phenol–chloroform method. A PacBio library (15–20 kb) was prepared using the SMRTbell Express Template Prep Kit 2.0. Single-molecule real-time sequencing was carried out on a PacBio Sequel II platform under the CCS mode. Then, the subreads were filtered by minimum length of 50 kb, and the HiFi reads were generated using ccs software (v 4.2.0) (https://github.com/PacificBiosciences/ccs) with the parameters of “min-passes = 3, min-rq = 0.99”. For genome survey, Illumina library was constructed using the DNA from the same oyster, and sequenced by a PE150 strategy on Illumina NovaSeq 6000 system. For Hi-C sequencing, the adductor muscle from the same individual for DNA extraction was fixed with 1% formaldehyde, and DNA was cross-linked and digested with MboI restriction enzyme. The library was also sequenced on an Illumina NovaSeq 6000 platform with a PE150 method.

### Genome survey, assembly, and scaffolding

Genome survey was performed using k-mer frequency-based method. First, the Illumina reads was trimmed and filtered with fastp (v 0.23.1) [78]. The k-mers were then counted using Platanus-allee (v 2.2.2) [79]. Finally, the output file was used as the input for GenomeScope (v 2.0) [80] to estimate the genome size, rate of heterozygosity, and abundance of repetitive elements. The initial genome was de novo assembled with hifiasm (v 0.15.1-r328) [81]. The Hi-C sequencing reads were mapped to the contigs by Burrows-Wheeler Aligner (BWA) (v 0.7.17-r1198-dirty) [82]. Then, Juicer (v 1.6) [83] was used for the construction of Hi-C contact matrix, and the anchoring was performed with 3D-DNA (v 180,419) [84]. Finally, the Juicebox Assembly Tools (v 1.11.08) [85] were applied for the manual correction of the connections.

### Quality assessment of genome assembly

To assess the quality of genome assembly, BUSCO (v 5.1.2) [86] analysis was used to verify the completeness of genome. QUAST (v 5.0.2) [87] was used to check genome assembly quality with the raw PacBio HiFi reads. In addition, Illumina pair-end reads were mapped back to the assembly with BWA (v 0.7.17-r1198-dirty) [82]. Mapping statistics were summarized with samtools software (version 1.15) [88].

### Repeat annotation

Repetitive sequences were identified and masked using both homology and de novo predictions. Briefly, RepeatModeler (v 2.0.1) [89] was used to construct a de novo repeat library. The consensus sequences in de novo repeat library were further combined with molluscan repetitive sequences from both Repbase library (v 20,181,026) (https://www.girinst.org/repbase/) and Dfam database (v 3.3) [90], and then used to run RepeatMasker (v 4.1.2-p1) [91] on the genome assembly. TE (transposable element) divergence analysis was performed using an R script (https://github.com/ValentinaBoP/Transposable Elements) with the detailed annotation table from the output of RepeatMasker software.

### Gene structure and functional annotation

Transcriptome alignment, de novo prediction, and homology-based methods were combined to predict protein-coding genes in *C. nippona* genome. For transcriptome-based prediction, total RNA was respectively isolated from seven tissues of the same oyster used in genome sequencing (including adductor muscle, digestive gland, gill, male gonad, hemolymph, labial palp, and mantle) as well as female gonad tissue from another wild individual, using TRIzol reagent according to the manufacturer’s instructions. RNA samples from all tissues were pooled (in equal amounts) and used for full-length sequencing on a PacBio Sequel II platform. Raw reads were processed using SMRT Link software (v 9.0) (https://www.pacb.com/support/software-downloads). In addition, RNA-seq short reads generated in our previous research [92] were downloaded from NCBI SRA database (SRR10482020, SRR10482021, SRR10482022, SRR7646736). These downloaded data were pre-processed by fastp (v 0.23.1) [78] and assembled following PASA pipeline (v 2.4.1) [93]. High-quality full-length transcripts generated from SMRT Link software and assembled transcripts from PASA were further clustered with cd-hit-est (v 4.8.1) [94]. For de novo gene prediction, Augustus (v 3.4.0) [95] was trained by Braker2 (v 2.1.5) [96] with short RNA-seq reads. For homologous annotation, protein sequences of *C. gigas*, *C. virginica*, *M. yessoensis*, *Aplysia californica*, and *Octopus bimaculoides* were downloaded from NCBI database. Moreover, manually annotated protein sequences (> 50aa) of Bivalvia were obtained from the Uniprot/Swiss-Prot database (Release 2022_1). Finally, a high confidence gene set was generated using Maker (v 3.01.03) [97] with the trained Augustus predictor, transcript sets, and protein sequences from NCBI and Uniprot/Swiss-Prot databases.

Functional annotation of protein-coding genes was carried out by comparing alignments to public databases including NCBI non-redundant (NR), Uniprot/Swiss-Prot, EggNOG (v 5.0) [98], Pfam (Pfam-A v 35.0) [99], GO categories, and Kyoto Encyclopedia of Genes and Genomes (KEGG) pathways [100]. Gene motifs and domains were also identified using InterProScan (v 5.52–86.0) [101]. For the annotation of ncRNA, the tRNAscan-SE (v 2.0.7) [102] was employed to predict tRNAs. Screens for rRNAs, miRNAs, and snRNAs were performed using the INFERNAL (v 1.1.2) [103] against Rfam database (v 14.5) [104].

### Gene family and phylogenetic analyses

The ortholog groups (OGs) of 16 molluscan protein sets were identified using OrthoFinder (v 2.5.2) [105]. Multiple protein sequence alignments were performed with MAFFT (v 7.475) [106] under default parameters. OGs from selected molluscan taxa were used for subsequent phylogenomic analysis. Phylogenetic tree was constructed based on a total of 1253 one-to-one single-copy orthologous genes by FastTree 2 within OrthoFinder (v 2.5.2) [105]. The MCMCTree [107] was used to predict the divergence time among the selected species with nine calibration points (Additional file 2: Table S7) obtained from TimeTree database [108]. Expansion and contraction of gene families was estimated using by CAFE (v 5) [109] on the basis of the results from OrthoFinder software (v 2.5.2) and species divergence time. Gene families with *P* value less than 0.05 were considered as an event of significant expansion or contraction.

To identify tyrosinase, peroxidase, TIMP, VWA, CB, and LG domains, the hmmsearch software was first used to search against the PFAM domain (PF00264.23, PF03098.18, PF00965.20, PF00092.31, PF01607.27, and PF13385.9, respectively) with an E-value threshold of 1e − 5. Then, we used InterProScan (v 5.52–86.0) [101] against SMART, Pfam, and SUPERFAMILY databases. Molecular phylogenetic analyses were respectively conducted using tyrosinase, peroxidase, and TIMP domain-containing proteins that were identified from 15 protostomian genomes (Additional file 2: Table S14) by hmmsearch and InterProScan. Sequence alignment was performed using the program MAFFT (v 7. 475) [106]. The ML (maximum likelihood) phylogenetic trees were constructed using IQ-Tree (v 2.1.4-beta) [110] with 1000 bootstraps. The final trees were visualized and labeled using iTOL (v 6.7) online (https://itol.embl.de/). For Pif proteins, the phylogenetic tree was built using identified proteins from 16 metazoan genomes (Additional file 2: Table S14) following the pipeline described above.

### Synteny analysis

MCscanX in the JCVI toolkit (v 1.1.12) (https://github.com/tanghaibao/jcvi) [111] was used to identify and visualize macro-synteny. We analyzed chromosome collinearity between *C. nippona* and the other three oysters (*C. gigas*, *C. ariakensis*, and *C. virginica*). In addition, syntenic analysis was also performed among *C. nippona*, *M. yessoensis* [112], and *P. fucata* [113].

### Transcriptome sequencing and analysis

Farmed *C. nippona* individuals (3-year-old) were collected from the oyster farm of Rushan, Shandong Province, China. For shell regeneration experiment, holes were drilled in the centers of left shells of three oysters. During experiment, shell-damage oysters were cultured in a tank with seawater (seawater temperature of 22 ± 2 ℃ and salinity of 30 ppt) and fed with *Chlorella* sp. daily. ME and MC were sampled from left valves of drilled oysters at 10 days post drilling. The other three non-drilled oysters were dissected into adductor muscle, digestive gland, gill, hemolymph, ME, and MC. Notably, it is difficult to differentiate the distinct regions of the mantle by naked eye. Thus, the dissected ME may contain the outer zone of the mantle (outer pallium and mantle edge), while the MC represents the inner zone of the mantle (inner pallium).

Total RNA was extracted using TRIzol and further sequenced in PE150 mode on an Illumina NovaSeq 6000 platform to produce ~ 6 Gb data for each tissue sample. In addition, RNA-seq data from different tissues of *C. gigas*, *Ostrea edulis*, *P. fucata*, *M. yessoensis*, *Mercenaria mercenaria*, and *N. pompilius* were downloaded from NCBI (Additional file 2: Table S15). The raw reads of seven species were quality-filtered with fastp (v 0.23.1) [78], and then mapped to their own genomes using HISAT2 (v 2.2.1) [114]. For each species, the expression levels of genes were calculated with featureCounts (v 2.0.1) [115] and normalized using transcripts per million mapped reads (TPM) and trimmed mean of M-values (TMM). The DEGs for each tissue were identified with a Trinity utilities script on default parameters using edgeR software package (v 3.40.2) [116]. Tissue-specific genes were determined on the basis of their expression levels compared across all tissue types. Specifically, the mantle-specific genes of *C. nippona* were identified with both the mantle edge and central mantle samples against other tissue groups. Only genes which were overexpressed with log_2_(fold change) > 1 and false discovery rate (FDR) < 0.05 against other tissue types were classified as highly expressed genes.

### Real-time PCR validation

To validate our RNA-seq data, quantitative real-time PCR was conducted on selected genes which are highly expressed in the mantle of *C. nippona*, using elongation factor 1-alpha (EF1a) as the internal standard gene. The primers were designed with Primer 6.0 software (Additional file 2: Table S16). Real-time PCR was performed with QuantiNova™ SYBR® Green PCR Kit following the instruction manual of the kit (QIAGEN) on a LightCycler 480 real-time PCR system (Roche). All primer pairs for the PCR amplification were checked by the melting curve method. Three biological replicates for each tissue type were guided. The comparative cycle threshold (Ct) method was applied to quantify the relative expression levels based on the 2^−ΔΔCt^ method [117].

### In situ* hybridization*

Antisense probes were synthesized using purified PCR products (1 μg per reaction) (Additional file 2: Table S16) and DIG RNA labeling Kit (T7) (Roche), following the manufacturer’s instructions. Probe synthesis reactions were performed at 37 °C for 3 h and then were treated with DNase I (Promega) at 37 °C for 20 min. Synthesized probes were purified using the MEGAclear™ Transcription Clean-Up Kit (Thermo Fisher Scientific) and stored at − 80℃. Mantle tissues of *C. nippona* were fixed in 4% PFA solution overnight at 4 °C. Then, samples were dehydrated with serial methanol (25, 50, 75, and 100%) and stored at − 20℃.

In situ hybridization of mantles was carried out according to the methods as described previously [118] with slight modifications. Briefly, the fixed mantles were transferred to methanol, cleared in xylene, embedded in paraffin wax, and cut into 5-μm-thick sections on a Leica RM 2016 rotary microtome (Leica). After a series of deparaffinization, hydration, digestion, prehybridization, hybridization (final concentration of RNA probes: 1 ng/μl), and antibody incubation (with a 1:3000 dilution of antiDIG-AP antibody in the blocking buffer), sections were incubated with 2% NBT/BCIP solution (Roche) in darkness at 4 °C overnight. Finally, pictures were taken under an Olympus BX53 microscope coupled with a DP80 camera (Olympus).

### Scanning electron microscopy (SEM)

To characterize crystal structures, the *C. nippona* shells were fractured and carefully collected with a dissecting knife under an anatomical lens. After a 5-min ultrasonic cleaning, the shells were dried and sputter-coated with a thin layer of gold nanoparticles. Then, the surfaces and vertical sections of shells were scanned using the VEGA3 TESCAN scanning electron microscope.

### LC–MS/MS analysis

Fresh shells of six *C. nippona* individuals were incubated in 1% sodium hypochlorite (NaOCl) for 24 h and mechanically washed in the Milli-Q water to remove remaining tissues, superficial epibionts, and periostracum. The outer prismatic, inner foliated, and chalky layers were identified by their color and carefully separated using a dissecting knife. Separated shell layers were cleaned with a 5-min ultrasound treatment in the Milli-Q water and then air-dried at room temperature (RT).

The cleaned shell layers were roughly crushed into fine powder and treated with SDT-lysis buffer (4% SDS, 100 mM DDT, 100 mM Tris–HCl, pH 7.6) in a boiling water bath for 5 min. The SMPs in this study were extracted from soluble shell matrix. After cooling to RT, the supernatant was collected by a short centrifugation, then mixed with UA buffer (8 M Urea, 150 mM Tris–HCl, pH 8.0). The mixture was ultra-filtered on 10 kDa cut-off membrane, and alkylation was performed with 50 mM iodoacetamide in UA buffer for 30 min at RT in the dark. After washing with UA buffer and NH_4_HCO_3_ solution sequentially, samples were digested with trypsin solution (6 µg trypsin in 40 µl NH_4_HCO_3_ buffer) at 37 °C for 16 h, desalted via C18 Stage Tips and dried off in a vacuum concentrator. The dried peptides were then reconstituted in 0.1% formic acid for analysis by a Q-Exactive Plus mass spectrometer coupled to an EASY-nLC 1200 system (Thermo Fisher Scientific).

Peptide fragments were analyzed against the predicted gene models of *C. nippona* using the intensity-based absolute quantification (iBAQ) method in MaxQuant (v 1.6.17.0) [119]. Minor and major proteins were discerned following the procedures as previously described [120]. In addition, amino acid sequences of minor proteins were searched against SMP database (https://doi.org/10/cz2w) [56] and shell proteome of *C. gigas* [30], using BLASTP (v 2.11.0) with an E-value of 1e − 100 and sequence identity of 80%. Major proteins and the best matches of minor proteins were identified as SMPs of *C. nippona* in this study. Furthermore, SMPs of the other six molluscs including *C. gigas* [30], *Atrina pectinata* [15], *Tridacna crocea* [121], *P. fucata* [30], *Lottia gigantea* [122], and *N. pompilius* [49] were download and used for comparative analysis of shell proteomes. Functional domain annotations of SMPs were performed by searching against various databases, including SMART, CDD, Pfam, PROSITE patterns, PROSITE profiles, and SUPERFAMILY, using InterProScan (v 5.52–86.0) [101]. The signal domains of proteins were identified with SignalP-6.0 [123].

### Supplementary Information

**Additional file 1: Figure S1. **Genome assembly of *C. nippona*. **Figure S2.** Distribution of TEs in the *C. nippona* genome. **Figure S3. **Genomic synteny between *C. nippona* and other molluscs. **Figure S4. **Expansion of tyrosinase gene family in Protostomia. **Figure S5.** Expansion of peroxidase gene family in Protostomia. **Figure S6. **Expansion of tissue inhibitor of metalloproteinase (TIMP) gene family in Protostomia. **Figure S7. **Real-time PCR results showing gene expression patterns among tissues of *C. nippona*. **Figure S8. **Ultrastructure of the *C. nippona* shell. **Figure S9.** Base peak chromatogram of three types of protein sample of the *C. nippona* shell. **Figure S10. **Expression patterns of genes encoding shell matrix proteins (SMPs) in six types of tissues of *C. nippona*.** Figure S11.** Protein-domain analysis of shell proteomes from seven molluscs. **Figure S12. **Cartoon representation indicating domain structures of four SMPs in Figure 2e. **Figure S13. **Observation of the shell repair process of *C. nippona*. **Figure S14. **SEM images representative of the ultrastructure of repaired shell of *C. nippona*. **Figure S15. **Flower plot comparing orthologous groups among seven species. **Figure S16. **Cartoon representation indicating domains in Pif and LamG3 proteins identified as SMPs of C. nippona. **Figure S17. **Maximum likelihood (ML) tree of Pif and LamG3 in seven molluscs with transcriptome data. **Figure S18. **Genomic arrangement of Pif, ancestral Pif, and LamG3 genes in mollusks.** Figure S19. **Tissue expression patterns and protein domain structures of Pif_LamG3_cluster members in bivalves.** Figure S20. **Spatial expression patterns of Pif and LamG3 genes in *C. nippona* mantle.

**Additional file 2: Table S1. **The *de novo* assembly statistics of *C. nippona* genome. **Table S2.** Comparison of assembly statistics among 16 mollusc genomes. **Table S3. **Composition of repetitive sequences in the *C. nippona* genome. **Table S4.** Statistics of gene predictions of the *C. nippona* genome. **Table S5.** Functional annotation of *C. nippona* predicted genes. **Table S6.** Statistics of ncRNA annotation in *C. nippona*. **Table S7. **Divergent time points used in calibration. **Table S8. **GO enrichment of unique gene families in Ostreida. **Table S9.** GO enrichment of expanded gene families in in Ostreida. **Table S10.** Shell matrix proteins detected from the prismatic (P), foliated (F) and chalky layer (C) of *C. nippona*. **Table S11. **Differentially expressed genes of mantle (edge vs centre) of *C. nippona*. **Table S12.** Differentially expressed genes of mantle (edge vs centre) of *C. nippona* during shell repair. **Table S13. **Conserved orthogroups shared by the shell proteomes of seven molluscs. **Table S14.** Genomic resources used for molecular phylogenetic analyses of gene families. **Table S15.** Mapping statistics of transcriptome data used in this study. **Table S16.** Primers used for real-time PCR and ISH.

**Additional file 3. **Command line scripts used in present study.

## Data Availability

All raw genome and transcriptome sequencing data used for genome assembly and annotation have been deposited at NCBI under the BioProject accession PRJNA947686 [124]. The RNA-seq data from various tissue transcriptomes of *Crassostrea nippona* (SRR23950953-SRR23950959, SRR23950967, SRR23950970-SRR23950979) and shell repair experiment (SRR23950960-SRR23950965) were deposited in NCBI SRA database under PRJNA947922 project [125]. The shell proteomic data, genome assembly, and annotation have been deposited on Figshare (https://doi.org/10.6084/m9.figshare.22336354) [126]. The code commands used in this study are available in the supplementary file (Additional file 3.txt).

## Abbreviations

Aam: *Amphibalanus amphitrite*
Adi: *Acropora digitifera*
AM: Adductor muscle
Apl: *Acanthaster planci*
Bfl: *Branchiostoma floridae*
BUSCO: Benchmarking Universal Single-Copy Orthologs
BWA: Burrows-Wheeler Aligner
CB: Chitin binding
Cin: *Ciona intestinalis*
Cni: *Crassostrea nippona*
COI: Cytochrome oxidase I
Ct: Cycle threshold
D: Digestive gland
Dme: *Drosophila melanogaster*
Dre: *Danio rerio*
EF1a: Elongation factor 1-alpha
ELC: EGF-like domain-containing
G: Gill
GMC: Glucose-methanol-choline
GO: Gene Ontology
H: Hemolymph
HiFi-CCS: High-fidelity circular consensus sequencing
Hsa: *Homo sapiens*
iBAQ: Intensity-based absolute quantification
IF: Inner fold
KEGG: Kyoto Encyclopedia of Genes and Genomes
Lan: *Lingula anatina*
LCA: Last common ancestor
LC–MS/MS: Liquid chromatography tandem mass spectrometry
LG: Concanavalin A-like lectin/glucanase
Lgi: *Lottia gigantea*
MC: Central mantle
Mco: *Mytilus coruscus*
ME: Mantle edge
MF: Middle fold
ML: Maximum likelihood
Mme: *Mercenaria mercenaria*
Mya: Million years ago
Mye: *Mizuhopecten yessoensis*
NaOCl: Sodium hypochlorite
ncRNA: Non-coding RNA
Npo: *Nautilus pompilius*
NR: Non-redundant
Obi: *Octopus bimaculoides*
OF: Outer fold
OGs: Ortholog groups
Pau: *Phoronis australis*
Pec: *Paraescarpia echinospica*
Pfu: *Pinctada fucata*
RLCD: Repetitive low complexity domain.
RT: Room temperature
SMP: Shell matrix protein
SP: Signal peptide
TE: Transposable element
TMM: Trimmed Mean of M-values
TPM: Transcripts per million mapped reads
VWA: Von Willebrand factor type A
