# Supplementary material for: Multi-omic insights into the formation and evolution of a novel shell microstructure in oysters
Source: BMC Biol. 2023 Sep 29;21:204. doi: 10.1186/s12915-023-01706-y (PMC10543319; doi:10.1186/s12915-023-01706-y)
Supplement: Supplementary file 1 — Additional file 1: Figure S1. Genome assembly of C. nippona. Figure S2. Distribution of TEs in the C. nippona genome. Figure S3. Genomic synteny between C. nippona and other molluscs. Figure S4. Expansion of tyrosinase gene family in Protostomia. Figure S5. Expansion of peroxidase gene family in Protostomia. Figure S6. Expansion of tissue inhibitor of metalloproteinase (TIMP) gene family in Protostomia. Figure S7. Real-time PCR results showing gene expression patterns among tissues of C. nippona. Figure S8. Ultrastructure of the C. nippona shell. Figure S9. Base peak chromatogram of three types of protein sample of the C. nippona shell. Figure S10. Expression patterns of genes encoding shell matrix proteins (SMPs) in six types of tissues of C. nippona. Figure S11. Protein-domain analysis of shell proteomes from seven molluscs. Figure S12. Cartoon representation indicating domain structures of four SMPs in Figure 2e. Figure S13. Observation of the shell repair process of C. nippona. Figure S14. SEM images representative of the ultrastructure of repaired shell of C. nippona. Figure S15. Flower plot comparing orthologous groups among seven species. Figure S16. Cartoon representation indicating domains in Pif and LamG3 proteins identified as SMPs of C. nippona. Figure S17. Maximum likelihood (ML) tree of Pif and LamG3 in seven molluscs with transcriptome data. Figure S18. Genomic arrangement of Pif, ancestral Pif, and LamG3 genes in mollusks. Figure S19. Tissue expression patterns and protein domain structures of Pif_LamG3_cluster members in bivalves. Figure S20. Spatial expression patterns of Pif and LamG3 genes in C. nippona mantle. [file 12915_2023_1706_MOESM1_ESM.pdf]

Supplementary Materials

Multi-omic insights into the formation and evolution of a novel shell microstructure in oysters

Yitian Bai,[†,1] Shikai Liu,[†,1] Yiming Hu,[1] Hong Yu,[1] Lingfeng Kong,[1] Chengxun Xu,[1] and Qi Li*,[1,2]

[1]Key Laboratory of Mariculture, Ministry of Education, Ocean University of China, Qingdao 266003, China

[2]Laboratory for Marine Fisheries Science and Food Production Processes, Qingdao National Laboratory for Marine Science and Technology, Qingdao 266237, China

[†]These authors contributed equally to this work.

*Correspondence author: Qi Li (qili66@ouc.edu.cn)

(a)

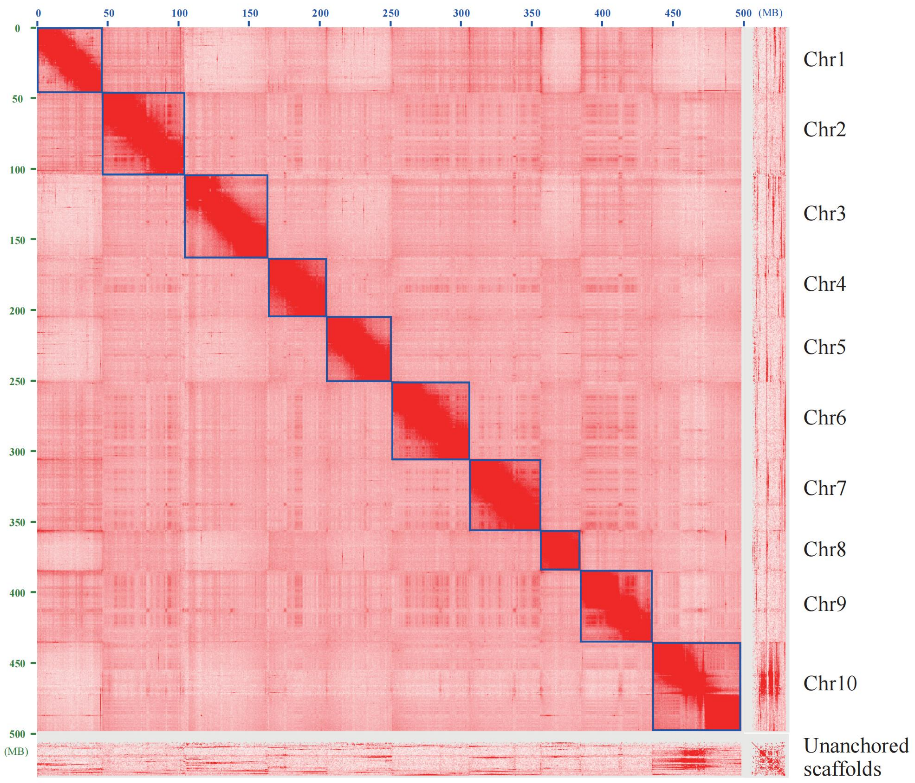

(b)

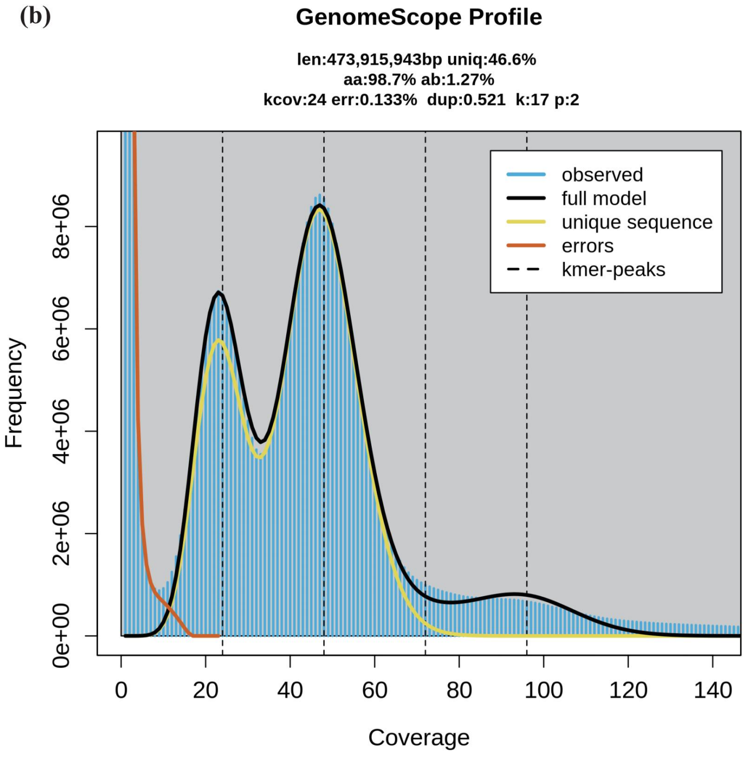

Figure S1. Genome assembly of *C. nippona*. (a) The Hi-C heatmap of genome assembly. The righ axis represents the chromosome number. (b) Estimated genome size of *C. nippona* based on K-mer analysis.

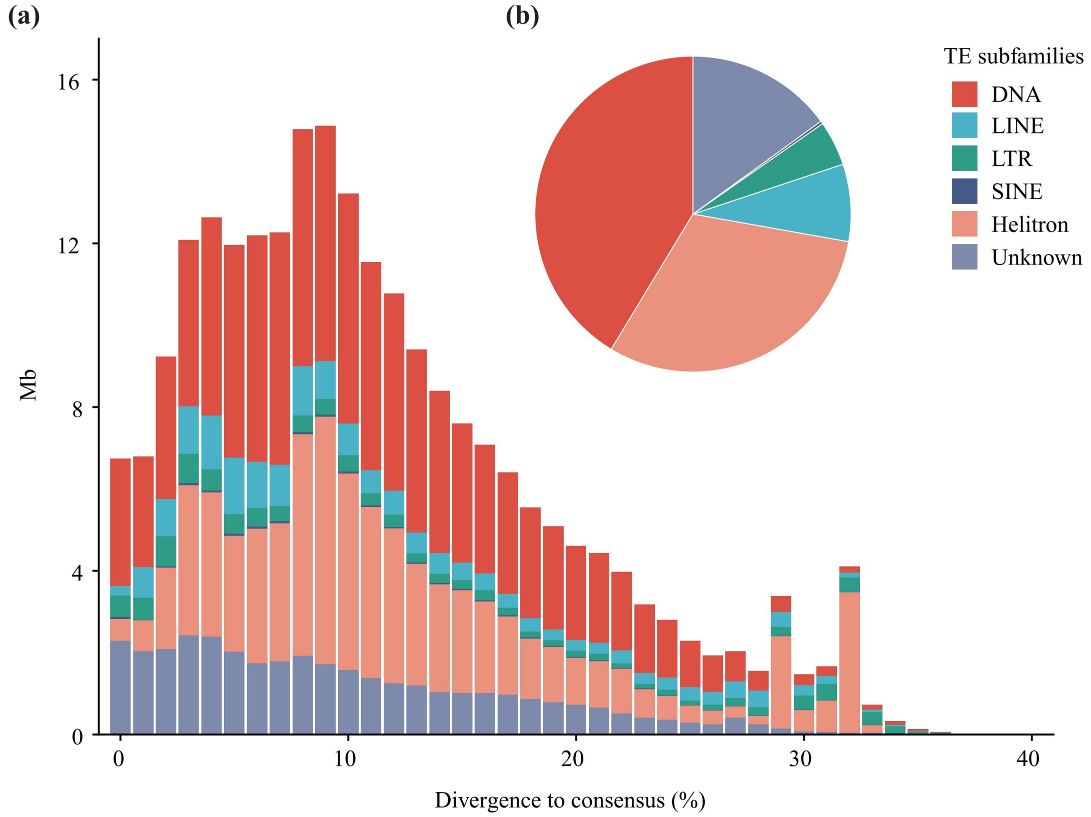

Figure S2. Distribution of TEs in the *C. nippona* genome. (a) History of TE accumulation in the *C. nippona* genome. Historical TE divergence was calculated by the Kimura distance-based copy divergence analysis. (b) Proportions of Helitrons, DNA transposons, LTR, LINE and SINE retrotransposons in the *C. nippona* genome.

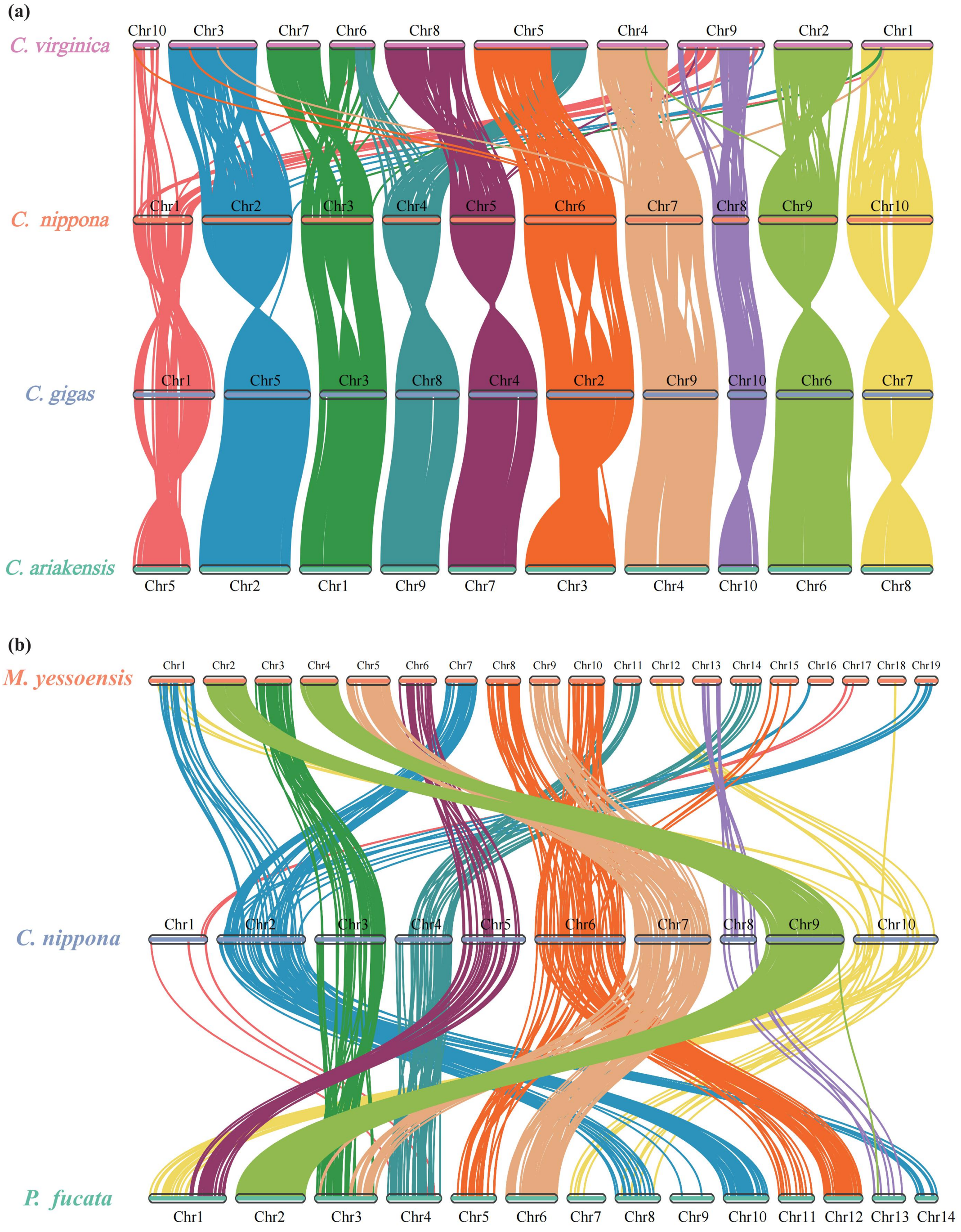

Figure S3. Genomic synteny between *C. nippona* and other molluscs. (a) Macro-synteny among four Crassostrea species. (b) Macro-synteny comparisons across *C. nippona*, *M. yessoensis*, and *P. fucata*.

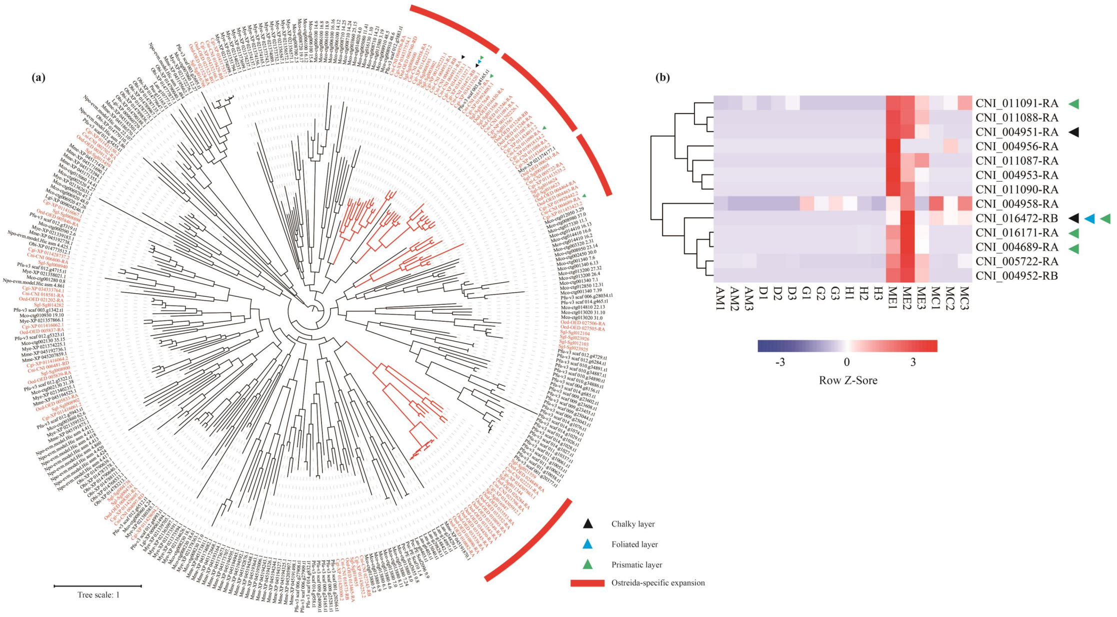

Figure S4. Expansion of tyrosinase gene family in Protostomia. The black, blue, and green triangles respectively represent the SMPs identified in the prismatic, foliated, and chalky layer. (a) Phylogeny of protostomian tyrosinases demonstrates oyster-specific expansions. (b) Expression pattern of oyster-specific expanded tyrosinase genes in *C. nippona*. The number after the abbreviation of tissue represents biologically independent individuals (N = 3). Abbreviations: AM, adductor muscle; D, digestive gland; G, gill; H, hemolymph; MC, mantle center; ME, mantle edge.

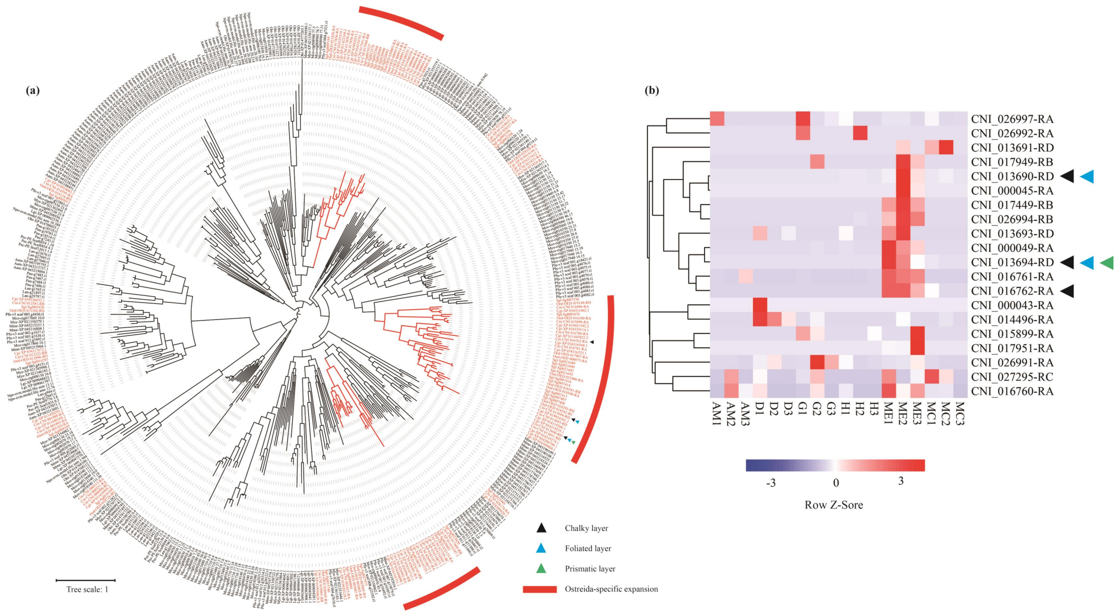

Figure S5. Expansion of peroxidase gene family in Protostomia. The black, blue, and green triangles respectively represent the SMPs identified in the prismatic, foliated, and chalky layer. (a) Phylogeny of protostomian peroxidases demonstrates oyster-specific expansions. (b) Expression pattern of oyster-specific expanded peroxidase genes in *C. nippona*. The number after the abbreviation of tissue represents biologically independent individuals (N = 3). Abbreviations: AM, adductor muscle; D, digestive gland; G, gill; H, hemolymph; MC, mantle center; ME, mantle edge.

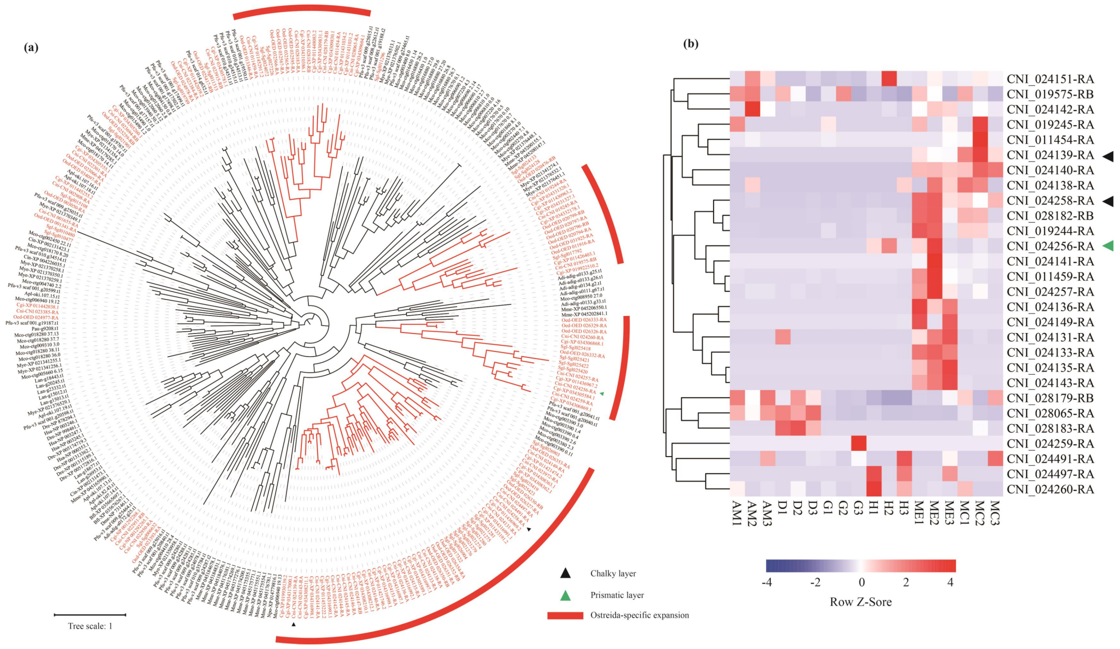

Figure S6. Expansion of tissue inhibitor of metalloproteinase (TIMP) gene family in Protostomia. The black and green triangles respectively represent the SMPs identified in the prismatic and chalky layer. (a) Phylogeny of protostomian TIMPs demonstrates oyster-specific expansions. (b) Expression pattern of oyster-specific expanded TIMP genes in *C. nippona.* The number after the abbreviation of tissue represents biologically independent individuals (N = 3). Abbreviations: AM, adductor muscle; D, digestive gland; G, gill; H, hemolymph; MC, mantle center; ME, mantle edge.

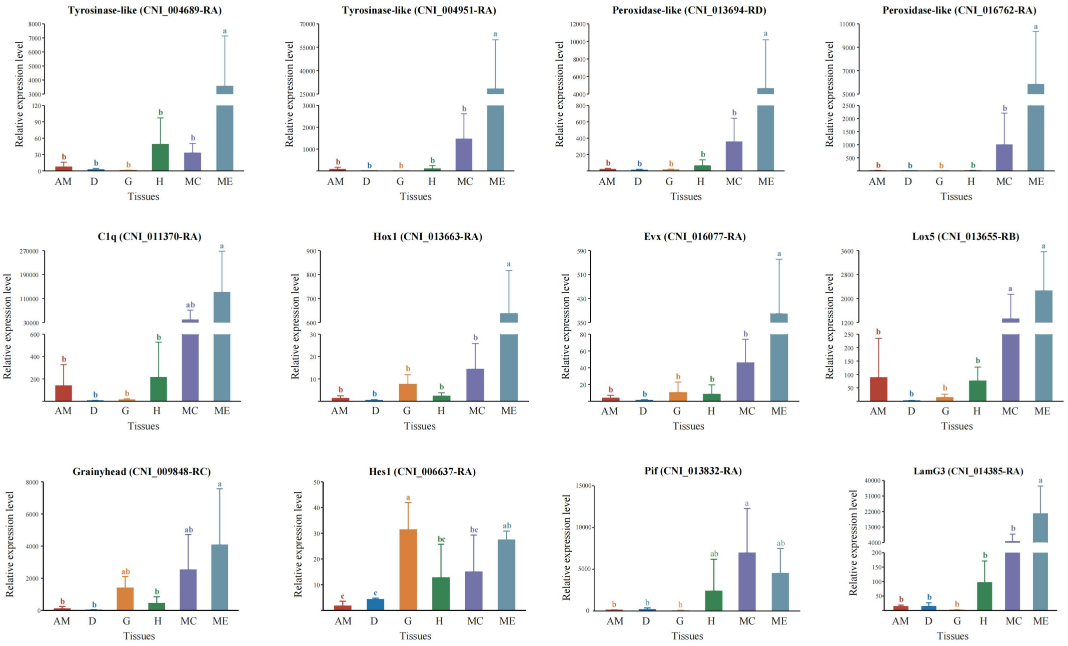

Figure S7. Real-time PCR results showing gene expression patterns among tissues of *C. nippona* (N = 3 biologically independent individuals). Statistical significance ($P < 0.05$) shown by different letters was determined using the LSD test in the R package agricolae (v 1.3-5).

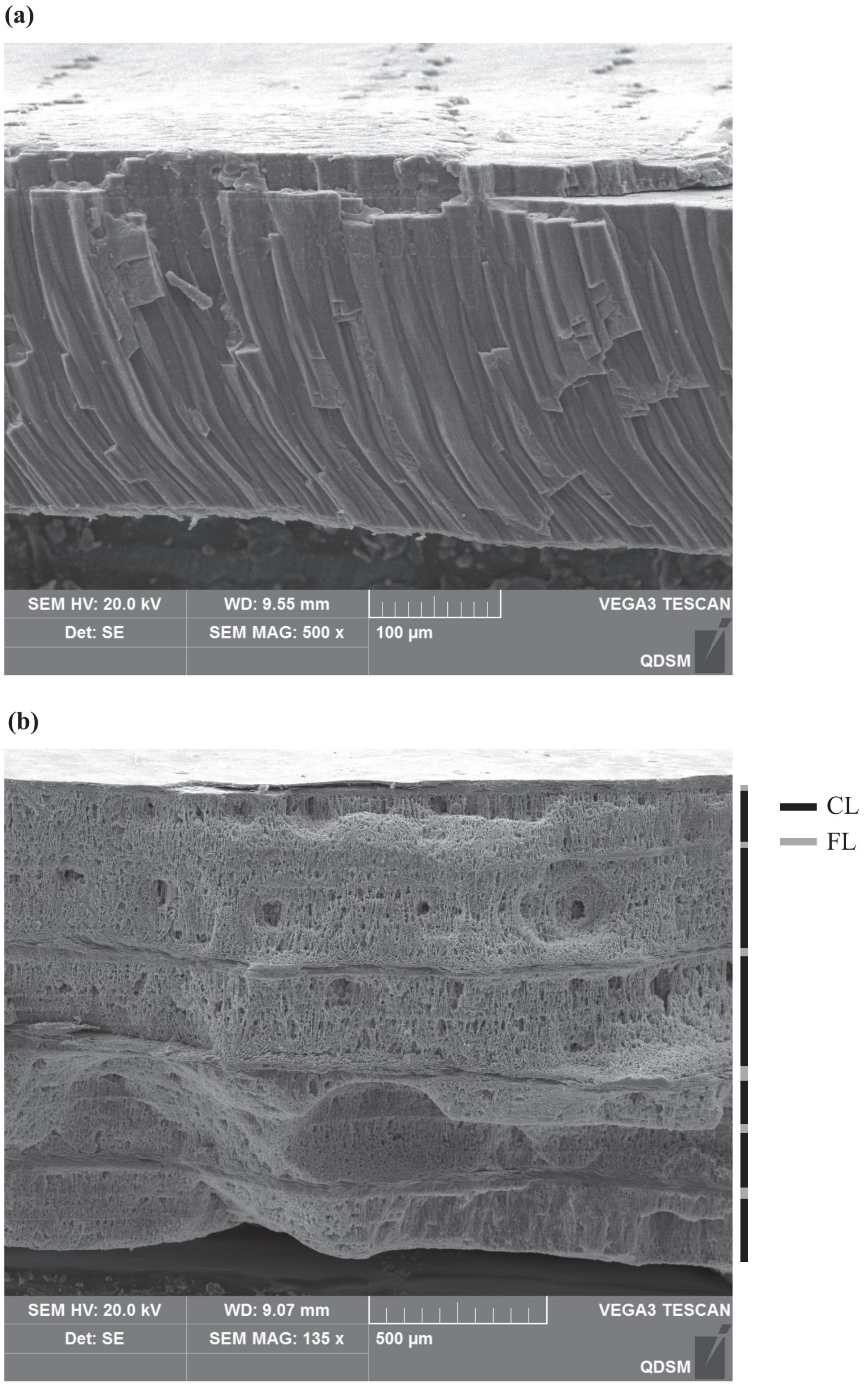

Figure S8. Ultrastructure of the *C. nippona* shell. (a) Outer layer of prisms. (b) Inner multi-layered structures. Abbreviations: FL, foliated layer; CL, chalky layer.

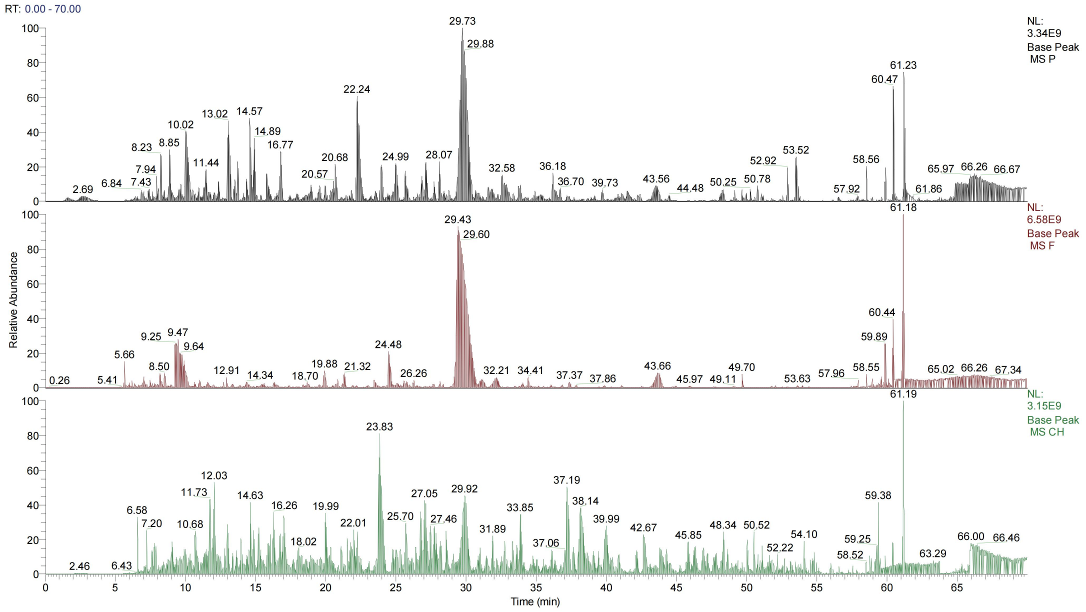

Figure S9. Base peak chromatogram of three types of protein sample of the *C. nippona* shell. Black color indicates protein from the prismatic layer; Red color: foliated layer; Green color: chalky layer.

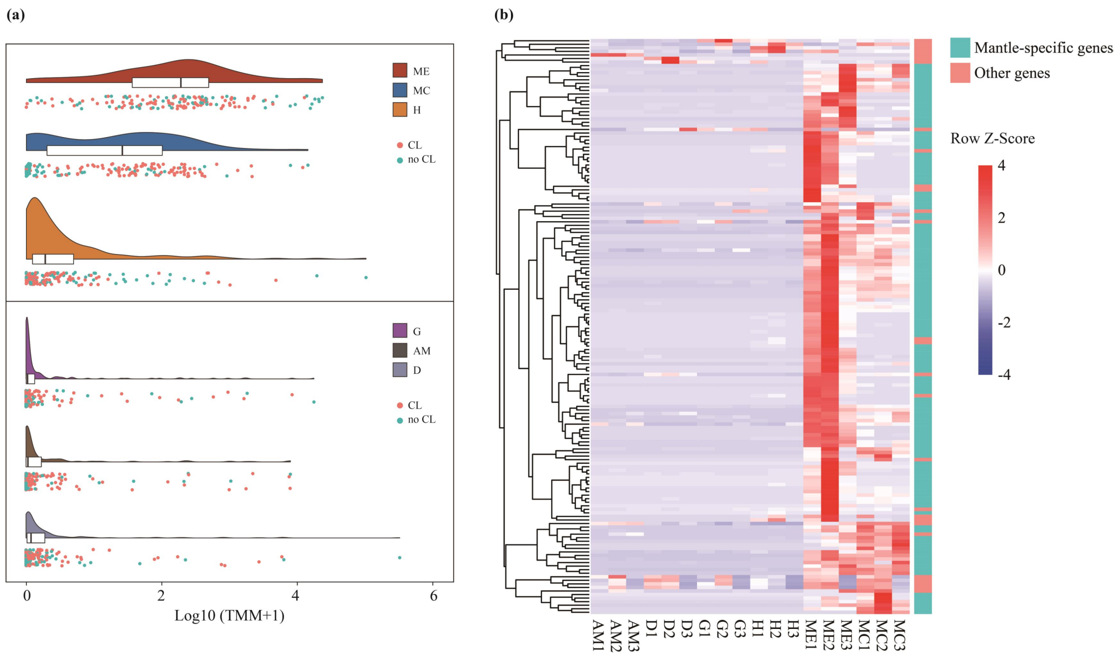

Figure S10. Expression patterns of genes encoding shell matrix proteins (SMPs) in six types of tissues of *C. nippona*. Abbreviations: AM, adductor muscle; D, digestive gland; G, gill; H, hemolymph; ME, mantle edge; MC, central mantle. (a) Distribution of expression levels of genes encoding SMPs in six types of tissues. Red dots indicate SMPs involved in the chalky layer. Blue dots indicate SMPs which are not identified in the chalky layer. (b) Tissue-specific expression of SMPs in *C. nippona*. Heatmap shows the normalized expression profiles of SMPs in different tissues. Mantle-specific genes encoding SMPs are marked with green color on the right, while other genes are marked in dark pink. The number after the abbreviation of tissue represents biologically independent individuals (N = 3).

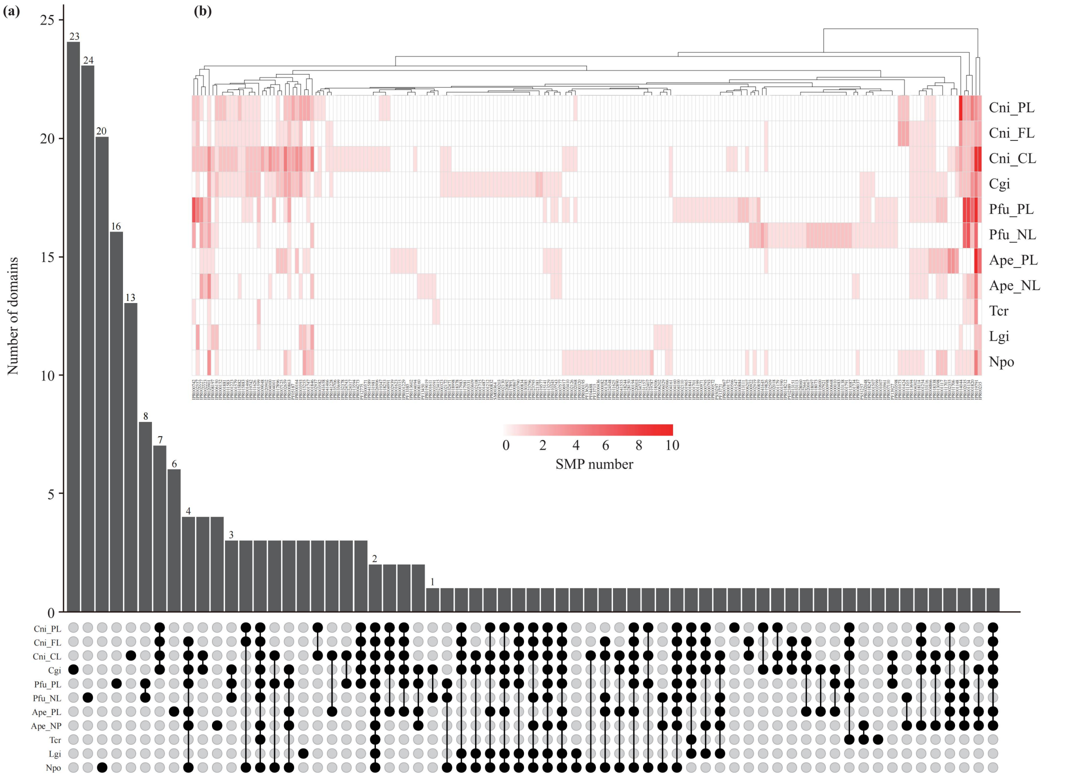

Figure S11. Protein-domain analysis of shell proteomes from seven molluscs, including *Crassostrea nippona* (Cni), *C. gigas* (Cgi), *Pinctada fucata* (Pfu), *Atrina pectinate* (Ape), *Tridacna crocea* (Tcr), *Lottia gigantea* (Lgi), *Nautilus pompilius* (Npo). Shell layers: prismatic layer (PL), foliated layer (FL), chalky layer (CL), nacre layer (NL). (a) Complete result of comparing the domains identified from the shell proteomes. The bar chart indicates the number of functional domains conserved among shell microstructure(s) or the whole shell of specie(s). (b) Distribution of SMP domains in different shell microstructures of seven species.

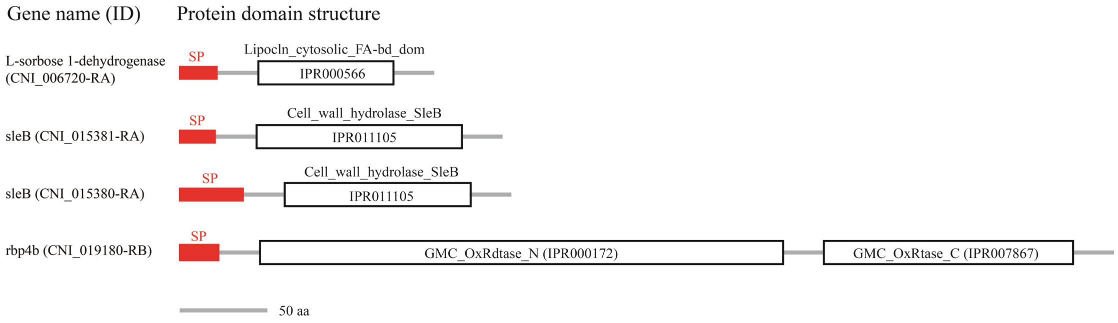

Figure S12. Cartoon representation indicating domain structures of four SMPs in Figure 2e. The red box represents signal peptide (SP). The unfilled box indicates the functional domain and the domain ID.

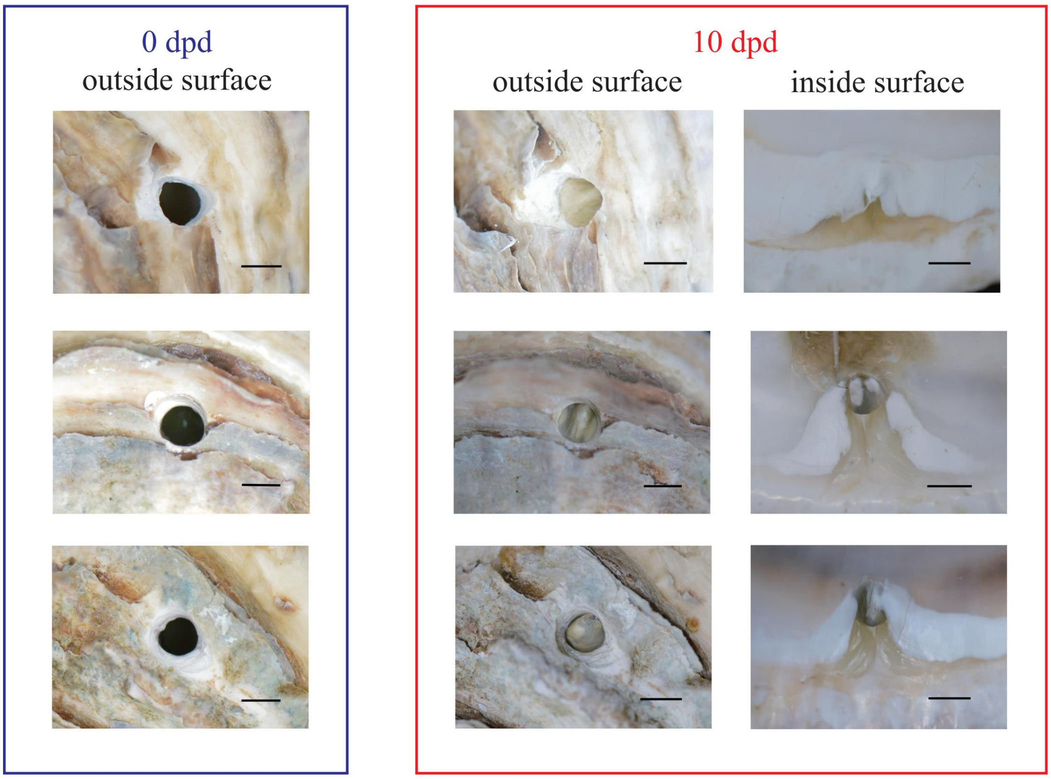

Figure S13. Observation of the shell repair process of *C. nippona*. dpd: days post shell-drilling. (scale bars: 5 mm)

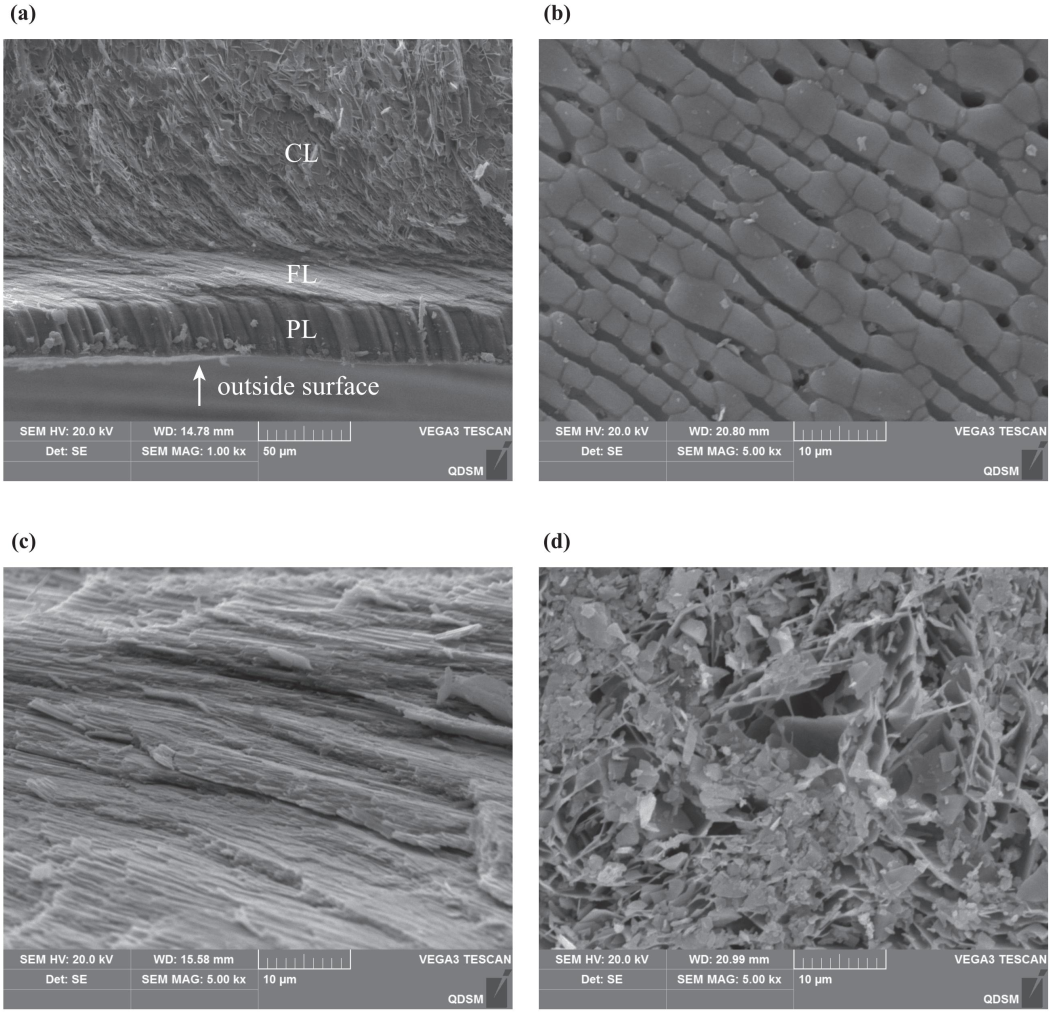

Figure S14. SEM images representative of the ultrastructure of repaired shell of *C. nippona*. (a) Cross section of the whole repaired shell. Abbreviations: PL, prismatic layer; FL, foliated layer; CL, chalky layer. (b) Repaired surface of PL. (c) Cross section of FL. (d) Cross section of CL.

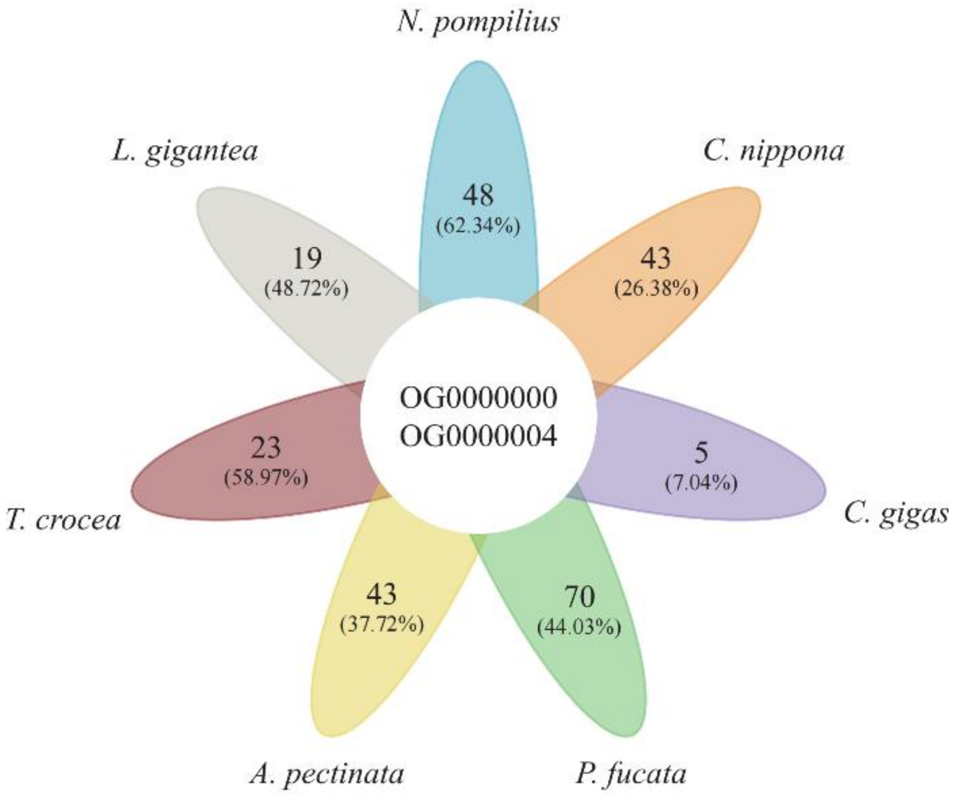

Figure S15. Flower plot comparing orthologous groups among seven species. Two conserved orthogroups are showed in the center of flower. Numbers show the species-specific SMPs of each species, while percentages in brackets indicate the proportion of species-specific SMPs in each species.

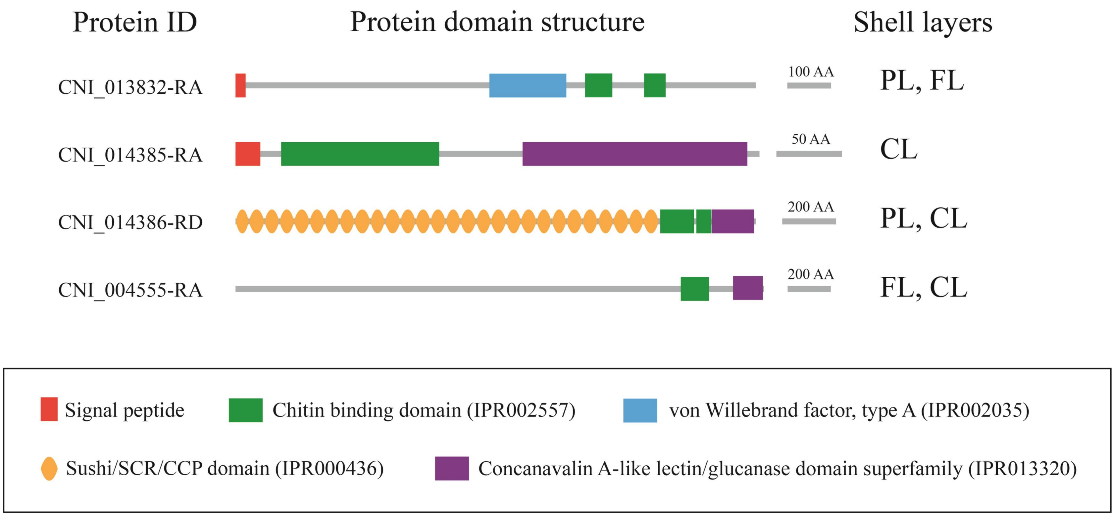

Figure S16. Cartoon representation indicating domains in Pif and LamG3 proteins identified as SMPs of *C. nippona*. Shell layers: prismatic layer (PL), foliated layer (FL), chalky layer (CL).

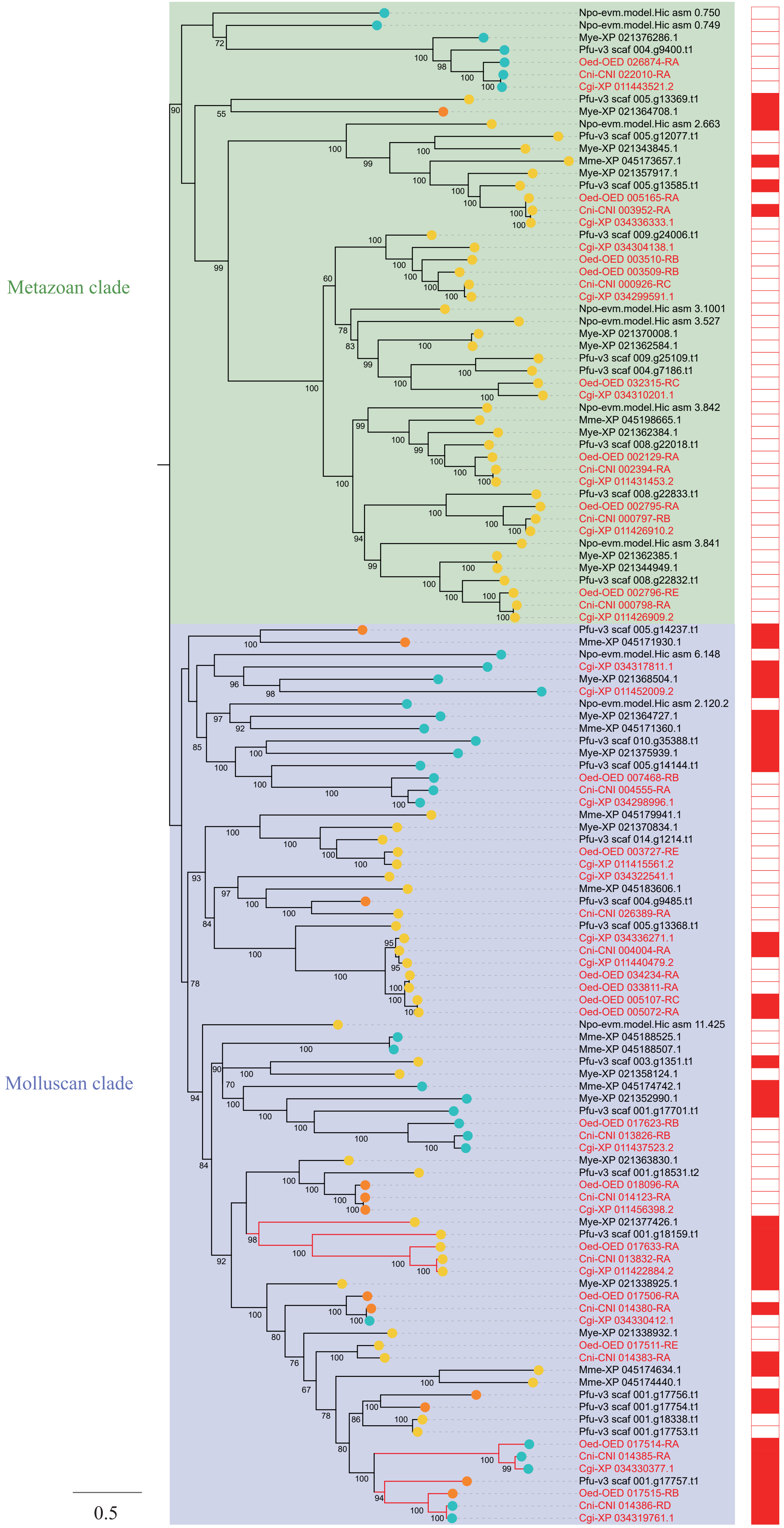

Metazoan clade
Molluscan clade
0.5
Npo-evm.model.Hic asm 0.750
Npo-evm.model.Hic asm 0.749
Mye-XP 021376286.1
Pfu-v3 scaf 004.g9400.t1
Oed-OED 026874-RA
Cni-CNI 022010-RA
Cgi-XP 011443521.2
Pfu-v3 scaf 005.g13369.t1
Mye-XP 021364708.1
Npo-evm.model.Hic asm 2.663
Pfu-v3 scaf 005.g12077.t1
Mye-XP 021343845.1
Mme-XP 045173657.1
Mye-XP 021357917.1
Pfu-v3 scaf 005.g13585.t1
Oed-OED 005165-RA
Cni-CNI 003952-RA
Cgi-XP 034336333.1
Pfu-v3 scaf 009.g24006.t1
Cgi-XP 034304138.1
Oed-OED 003510-RB
Oed-OED 003509-RB
Cni-CNI 000926-RC
Cgi-XP 034299591.1
Npo-evm.model.Hic asm 3.1001
Npo-evm.model.Hic asm 3.527
Mye-XP 021370008.1
Mye-XP 021362584.1
Pfu-v3 scaf 009.g25109.t1
Pfu-v3 scaf 004.g7186.t1
Oed-OED 032315-RC
Cgi-XP 034310201.1
Npo-evm.model.Hic asm 3.842
Mme-XP 045198665.1
Mye-XP 021362384.1
Pfu-v3 scaf 008.g22018.t1
Oed-OED 002129-RA
Cni-CNI 002394-RA
Cgi-XP 011431453.2
Pfu-v3 scaf 008.g22833.t1
Oed-OED 002795-RA
Cni-CNI 000797-RB
Cgi-XP 011426910.2
Npo-evm.model.Hic asm 3.841
Mye-XP 021362385.1
Mye-XP 021344949.1
Pfu-v3 scaf 008.g22832.t1
Oed-OED 002796-RE
Cni-CNI 000798-RA
Cgi-XP 011426909.2
Pfu-v3 scaf 005.g14237.t1
Mme-XP 045171930.1
Npo-evm.model.Hic asm 6.148
Cgi-XP 034317811.1
Mye-XP 021368504.1
Cgi-XP 011452009.2
Npo-evm.model.Hic asm 2.120.2
Mye-XP 021364727.1
Mme-XP 045171360.1
Pfu-v3 scaf 010.g35388.t1
Mye-XP 021375939.1
Pfu-v3 scaf 005.g14144.t1
Oed-OED 007468-RB
Cni-CNI 004555-RA
Cgi-XP 034298996.1
Mme-XP 045179941.1
Mye-XP 021370834.1
Pfu-v3 scaf 014.g1214.t1
Oed-OED 003727-RE
Cgi-XP 011415561.2
Cgi-XP 034322541.1
Mme-XP 045183606.1
Pfu-v3 scaf 004.g9485.t1
Cni-CNI 026389-RA
Pfu-v3 scaf 005.g13368.t1
Cgi-XP 034336271.1
Cni-CNI 004004-RA
Cgi-XP 011440479.2
Oed-OED 034234-RA
Oed-OED 033811-RA
Oed-OED 005107-RC
Oed-OED 005072-RA
Npo-evm.model.Hic asm 11.425
Mme-XP 045188525.1
Mme-XP 045188507.1
Pfu-v3 scaf 003.g1351.t1
Mye-XP 021358124.1
Mme-XP 045174742.1
Mye-XP 021352990.1
Pfu-v3 scaf 001.g17701.t1
Oed-OED 017623-RB
Cni-CNI 013826-RB
Cgi-XP 011437523.2
Mye-XP 021363830.1
Pfu-v3 scaf 001.g18531.t2
Oed-OED 018096-RA
Cni-CNI 014123-RA
Cgi-XP 011456398.2
Mye-XP 021377426.1
Pfu-v3 scaf 001.g18159.t1
Oed-OED 017633-RA
Cni-CNI 013832-RA
Cgi-XP 011422884.2
Mye-XP 021338925.1
Oed-OED 017506-RA
Cni-CNI 014380-RA
Cgi-XP 034330412.1
Mye-XP 021338932.1
Oed-OED 017511-RE
Cni-CNI 014383-RA
Mme-XP 045174634.1
Mme-XP 045174440.1
Pfu-v3 scaf 001.g17756.t1
Pfu-v3 scaf 001.g17754.t1
Pfu-v3 scaf 001.g18338.t1
Pfu-v3 scaf 001.g17753.t1
Oed-OED 017514-RA
Cni-CNI 014385-RA
Cgi-XP 034330377.1
Pfu-v3 scaf 001.g17757.t1
Oed-OED 017515-RB
Cni-CNI 014386-RD
Cgi-XP 034319761.1

Figure S17. Maximum likelihood (ML) tree of Pif and LamG3 in seven molluscs with transcriptome data (Additional file 2: Table S15). Pif and LamG3 genes of oysters are marked with red color. Yellow, orange, and blue solid circles represent Pif, ancestral Pif, and LamG3 genes, respectively. Red solid squares after gene IDs indicate the genes highly expressed in the mantle of selected molluscan species, while the red hollow squares indicate genes that are not highly expressed in the mantle. Red branches on the clades represent three groups of genes which are all highly expressed in the mantle tissue. Numbers on the nodes are bootstrap values (>50%).

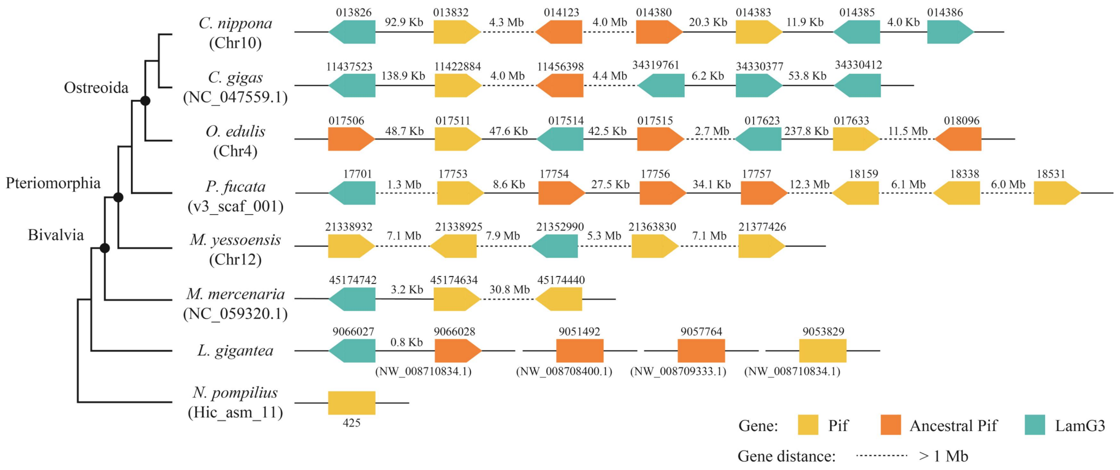

Figure S18. Genomic arrangement of Pif, ancestral Pif, and LamG3 genes in mollusks. Arrows indicate the direction of the transcripts. Dashed lines represent the long gaps (> 1 Mb) in the genome.

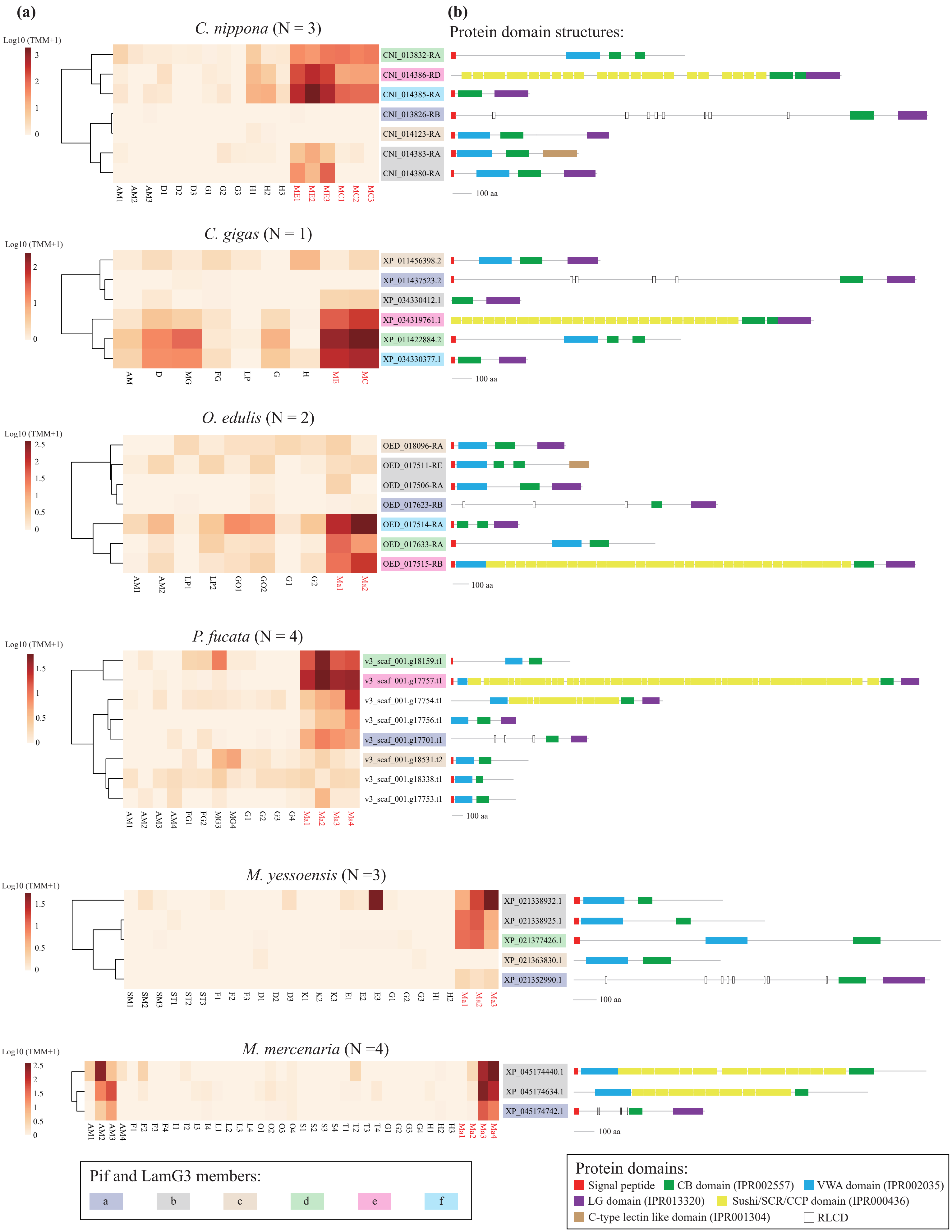
(a)
(b)
Protein domain structures:
C. nippona (N = 3)
Log10 (TMM+1)
3
2
1
0
CNI_013832-RA
CNI_014386-RD
CNI_014385-RA
CNI_013826-RB
CNI_014123-RA
CNI_014383-RA
CNI_014380-RA
AM1 AM2 AM3 D1 D2 D3 G1 G2 G3 H1 H2 H3 ME1 ME2 ME3 MC1 MC2 MC3
100 aa
C. gigas (N = 1)
Log10 (TMM+1)
2
1
0
XP_011456398.2
XP_011437523.2
XP_034330412.1
XP_034319761.1
XP_011422884.2
XP_034330377.1
AM D MG FG LP G H ME MC
100 aa
O. edulis (N = 2)
Log10 (TMM+1)
2.5
2
1.5
1
0.5
0
OED_018096-RA
OED_017511-RE
OED_017506-RA
OED_017623-RB
OED_017514-RA
OED_017633-RA
OED_017515-RB
AM1 AM2 LP1 LP2 GO1 GO2 G1 G2 Ma1 Ma2
100 aa
P. fucata (N = 4)
Log10 (TMM+1)
1.5
1
0.5
0
v3_scaf_001.g18159.t1
v3_scaf_001.g17757.t1
v3_scaf_001.g17754.t1
v3_scaf_001.g17756.t1
v3_scaf_001.g17701.t1
v3_scaf_001.g18531.t2
v3_scaf_001.g18338.t1
v3_scaf_001.g17753.t1
AM1 AM2 AM3 AM4 FG1 FG2 MG3 MG4 G1 G2 G3 G4 Ma1 Ma2 Ma3 Ma4
100 aa
M. yessoensis (N =3)
Log10 (TMM+1)
1.5
1
0.5
0
XP_021338932.1
XP_021338925.1
XP_021377426.1
XP_021363830.1
XP_021352990.1
SM1 SM2 SM3 ST1 ST2 ST3 F1 F2 F3 D1 D2 D3 K1 K2 K3 E1 E2 E3 G1 G2 G3 H1 H2 Ma1 Ma2 Ma3
100 aa
M. mercenaria (N =4)
Log10 (TMM+1)
2.5
2
1.5
1
0.5
0
XP_045174440.1
XP_045174634.1
XP_045174742.1
AM1 AM2 AM3 AM4 F1 F2 F3 F4 I1 I2 I3 I4 L1 L2 L3 L4 O1 O2 O3 O4 S1 S2 S3 S4 T1 T2 T3 T4 G1 G2 G3 G4 H1 H2 H3 Ma1 Ma2 Ma3 Ma4
100 aa
Pif and LamG3 members:
a
b
c
d
e
f
Protein domains:
Signal peptide
CB domain (IPR002557)
VWA domain (IPR002035)
LG domain (IPR013320)
Sushi/SCR/CCP domain (IPR000436)
C-type lectin like domain (IPR001304)
RLCD

Figure S19. Tissue expression patterns and protein domain structures of Pif_LamG3_cluster members in bivalves. (a) Expression heatmaps of Pif_LamG3_cluster members in different tissues of bivalves. Mantle tissues are marked by red font. The number after tissue name represents the biological duplicate. Abbreviations: AM, adductor muscle; D, digestive gland; E, eyes; F, foot; FG, female gonad; G, gill; GO, gonad; H, hemolymph; I, intestine; K, kidneys; L, liver; LP, labial palps; Ma, mantle; MC, mantle center; ME, mantle edge; MG, male gonad; O, ovary; S, stomach; SM, striated muscle (adductor muscle); ST, striated muscle (adductor muscle); T, testis. N means the number of biologically independent individuals. (b) Protein domain structures of Pif_LamG3_cluster members. RLCD means the repetitive low complexity domain.

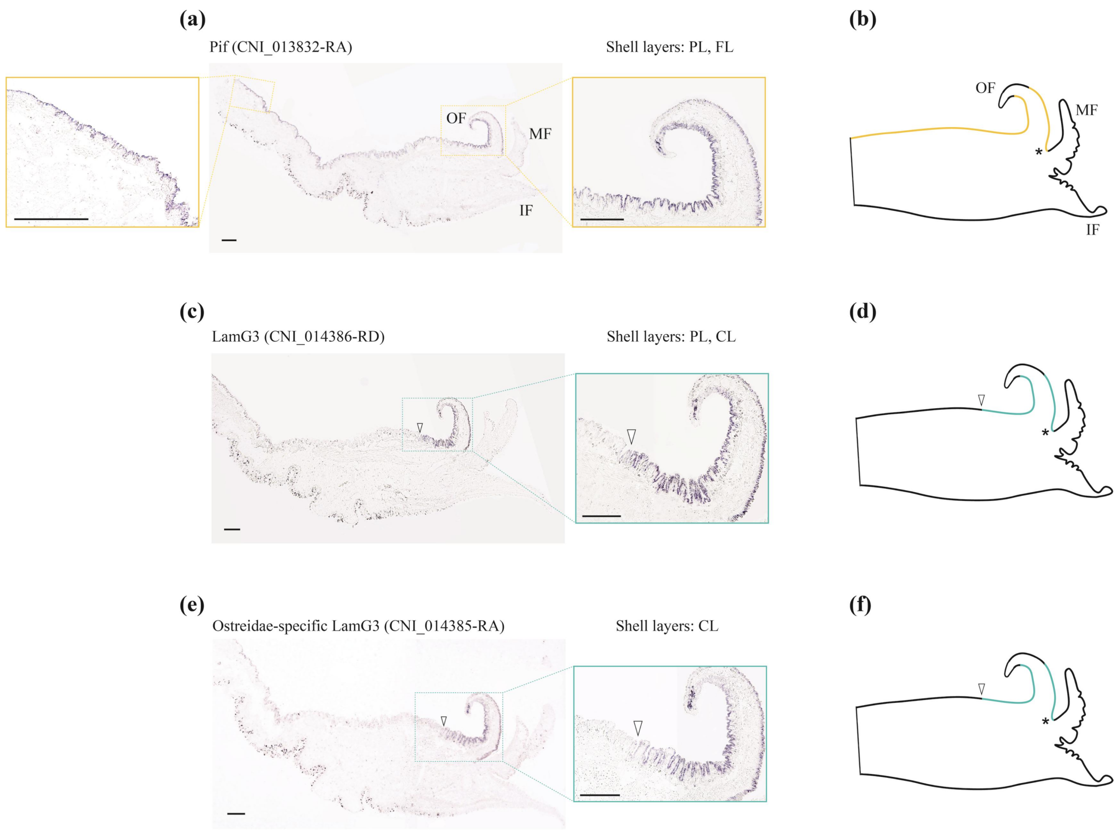

Figure S20. Spatial expression patterns of Pif and LamG3 genes in *C. nippona* mantle. White arrows symbolize the end of the gene expression region. Full view and partial enlargement show positive cells stained in blue by in situ hybridization of Pif (a), LamG3 (c), and Ostreidae-specific LamG3 (e) genes, respectively. Shell layers: prismatic layer (PL), foliated layer (FL), chalky layer (CL). Mantle folds: outer fold (OF), middle fold (MF), inner fold (IF) (scale bars: 200 μm). On the right side, the representative models of mantle show the spatial expression patterns of Pif (b), LamG3 (d), and Ostreidae-specific LamG3 (f) genes, respectively. Yellow color in the representative model of mantle indicates the expression regions of Pif (CNI_013832-RA), while blue color indicates the expression regions of LamG3 (CNI_014385-RA and CNI_014386-RA). Asterisks indicate the periostracal groove.
